# Drought has negative consequences on aphid fitness and plant vigor: Insights from a meta‐analysis

**DOI:** 10.1002/ece3.7957

**Published:** 2021-07-28

**Authors:** Daniel J. Leybourne, Katharine F. Preedy, Tracy A. Valentine, Jorunn I. B. Bos, Alison J. Karley

**Affiliations:** ^1^ Division of Plant Sciences School of Life Science Dundee University Dundee UK; ^2^ Ecological Sciences Department The James Hutton Institute Dundee UK; ^3^ Cell and Molecular Sciences Department The James Hutton Institute Dundee UK; ^4^ Biomathematics and Statistics Scotland Dundee UK

**Keywords:** climate change, ecological entomology, insect–environment interactions, meta‐analysis, plant–insect interactions

## Abstract

1. Aphids are abundant in natural and managed vegetation, supporting a diverse community of organisms and causing damage to agricultural crops. Due to a changing climate, periods of drought are anticipated to increase, and the potential consequences of this for aphid–plant interactions are unclear.
2. Using a meta‐analysis and synthesis approach, we aimed to advance understanding of how increased drought incidence will affect this ecologically and economically important insect group and to characterize any potential underlying mechanisms. We used qualitative and quantitative synthesis techniques to determine whether drought stress has a negative, positive, or null effect on aphid fitness and examined these effects in relation to (a) aphid biology, (b) geographical region, and (c) host plant biology.
3. Across all studies, aphid fitness is typically reduced under drought. Subgroup analysis detected no difference in relation to aphid biology, geographical region, or the aphid–plant combination, indicating the negative effect of drought on aphids is potentially universal. Furthermore, drought stress had a negative impact on plant vigor and increased plant concentrations of defensive chemicals, suggesting the observed response of aphids is associated with reduced plant vigor and increased chemical defense in drought‐stressed plants.
4. We propose a conceptual model to predict drought effects on aphid fitness in relation to plant vigor and defense to stimulate further research.

## Graphical abstract

Using meta‐analysis, we provide the first comprehensive analysis of drought stress on aphid fitness, showing negative effects across different host plant families, aphid tribes, and geographic regions. These negative effects were observed for different aphid fitness parameters and host specialisms and likely result from drought‐induced reduction in plant vigor and increased tissue concentrations of plant‐defensive compounds.

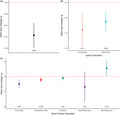

## 1 INTRODUCTION

The changing climate is anticipated to lead to decreased annual levels of precipitation in some regions, resulting in extended periods of drought (Blenkinsop & Fowler, 2007; Santos et al., 2016). For plants in mesic habitats, prolonged drought can have severe consequences on plant physiology, often leading to reduced growth and photosynthetic capacity (Osakabe et al., 2014; Zeppel et al., 2014). Plant physiological responses to drought can directly influence the population dynamics, fitness, phenology, and biology of herbivorous insects (Aslam et al., 2013; Huberty & Denno, 2004; Mody et al., 2009; Staley et al., 2006), with consequences that cascade through trophic networks (Johnson et al., 2011; Rodríguez‐Castañeda, 2013). Meta‐analysis provides a useful approach to predict the direction of drought effects on insect herbivores, reporting an overall response that accommodates between‐study variation.

Previous meta‐analyses have examined drought effects by comparing responses of herbivorous insect species with different feeding strategies (Huberty & Denno, 2004; Koricheva & Larsson, 1998). To date, however, there has been no comprehensive assessment of drought effects on a specific herbivore group and the underpinning causes due to physiological changes in the host plant. Aphids are phloem‐feeding insects of global ecological importance (Van Emden & Harrington, 2017) and are abundant components of insect communities in diverse ecosystems across the globe (Messelink et al., 2012; Roubinet et al., 2018). There are over 4,400 known species of aphid (Blackman & Eastop, 2000), and around 250 of these are major agricultural and horticultural pests, making them an economically important group of herbivorous insects. In many ecosystems, aphids sustain several higher trophic groups, including their primary consumers, such as parasitoid wasps, spiders, ladybirds, and carabid beetles (Staudacher et al., 2016); the higher‐level consumers of these aphid‐natural enemies, such as hyperparasitoids (Lefort et al., 2017; Traugott et al., 2008), small mammals, and birds; and many entomological pathogens and parasites (Hagen & van den Bosch, 1968). Examining how climate change, including drought, might influence aphid fitness is a major avenue of current research, specifically with regard to examining how this might affect the productivity and functioning of agricultural, horticultural, and natural vegetation systems across the globe (Romo & Tylianakis, 2013; Teixeira et al., 2020).

Analysis of sap‐feeding insects by Huberty and Denno (2004) suggested that drought has an overall negative effect on the fitness of sap‐feeding insects. Experimental studies of aphids indicate that this negative effect of drought is observed across many aphid–plant systems (Agele et al., 2006; Aslam et al., 2013; Foote et al., 2017; Grettenberger & Tooker, 2016; Mody et al., 2009; Pons & Tatchel, 1995), although this has not been assessed quantitatively. Further, there has been no comprehensive analysis of the causes of decreased aphid fitness under drought, although several studies suggest that it is mediated through reduced plant fitness (Banfield‐Zanin & Leather, 2015; Dai et al., 2015; Hale et al., 2003).

Drought can cause a reduction in plant vigor, an elevation in the concentration of plant‐defensive compounds, and an increase in nitrogen availability in leaf tissue (Cornelissen et al., 2008; Inbar et al., 2001; Ozturk et al., 2002; White, 1969); these physiological, nutritional, or chemical responses of plants to drought could potentially explain the response of aphids to drought. Two meta‐analyses conducted in recent decades provide context for constructing a hypothesis to explain variation in aphid fitness under water stress in relation to plant fitness. First, Huberty and Denno (2004) found little evidence for the plant stress hypothesis (i.e., enhanced insect performance on water‐stressed host plants due to increased tissue nitrogen availability; White, 1969) among sap‐feeding insects (phloem and mesophyll feeders). Second, Cornelissen et al. (2008) examined insect fitness in relation to plant vigor and demonstrated that sap‐feeding insects are more abundant and show increased fitness when feeding on more vigorously growing plants. These findings lead us to hypothesize that the effects of drought on aphid fitness are driven by decreased plant vigor, such as reduced plant growth rate or mass (Hatier et al., 2014), rather than stress‐related changes in plant nutritional quality.

Although many studies have reported reduced aphid fitness when exposed to drought‐stressed hosts (Banfield‐Zanin & Leather, 2015; Dai et al., 2015; Foote et al., 2017; Kansman et al., 2020), studies have reported null (Mewis et al., 2012) and positive (Oswald & Brewer, 1997) effects. Multiple factors could explain these contrasting observations, including differences in aphid or plant biology. Indeed, in the study by Oswald & Brewer a positive effect of drought on aphid fitness was detected in the Russian wheat aphid, *Diuraphis noxia* (Mordvilko), and a negative effect was reported for the corn leaf aphid, *Rhopalosiphum maidis* (Fitch). Although both species are cereal‐feeding aphids, *D. noxia* and *R. maidis* belong to two distinct aphid tribes, the Macrosiphini and the Aphidini, respectively (Choi et al., 2018; Kim & Lee, 2008), raising the possibility that differences in aphid biology and/or life history could underlie the contrasting responses. Additionally, the specific aphid–plant combination could further influence the effects of drought on aphid fitness. For example, multiple aphid species exhibit contrasting responses to drought on a common host plant (Mewis et al., 2012) and a single aphid species can display contrasting responses on related host plant species (Hale et al., 2003). These findings suggest that aphid responses to drought could be mediated by plant species‐specific responses to drought (i.e., the availability of nutrients, the concentration of defensive compounds, resource allocation to new tissue). Understanding these mechanisms is necessary to predict the outcomes of plant–insect interactions under a changing climate.

Here, we take the novel approach of analyzing data on both aphid fitness and host plant physiology, using studies comparing drought with unstressed conditions, to examine the hypothesis that changes in aphid fitness are driven by the effects of drought on plant vigor. We predicted that reduced aphid fitness would be associated more strongly with decreased plant vigor (e.g., decreased mass, reduced growth) under drought than with changes in plant nutritional quality or defensive chemistry. Initially, we carry out a literature synthesis and take a “vote‐counting” approach to qualitatively determine whether drought has an overall negative, positive, or null effect on aphid fitness. Following this, we use meta‐analysis techniques to quantify these effects. Next, we extract and analyze data reporting on plant physiological responses to drought, including measurements of plant vigor, and tissue concentrations of plant nutrients and plant‐defensive compounds. This provides us with data that can be used, for the first time, to quantify drought effects on plant physiology in parallel with aphid fitness responses. A secondary aim of the meta‐analysis was to explore patterns in aphid responses to drought in relation to (a) aphid biology, (b) geographic region, and (c) host plant biology (i.e., species combinations) to identify any common features that explain variation in aphid fitness responses to drought. The mechanistic understanding provided by our study allows the effects of drought on herbivore success to be anticipated for phloem‐feeding insects across different habitats under future climatic conditions.

## 2 MATERIALS AND METHODS

### 2.1 Literature search

#### 2.1.1 Search methodology and criteria for study inclusion

**FIGURE 1:**
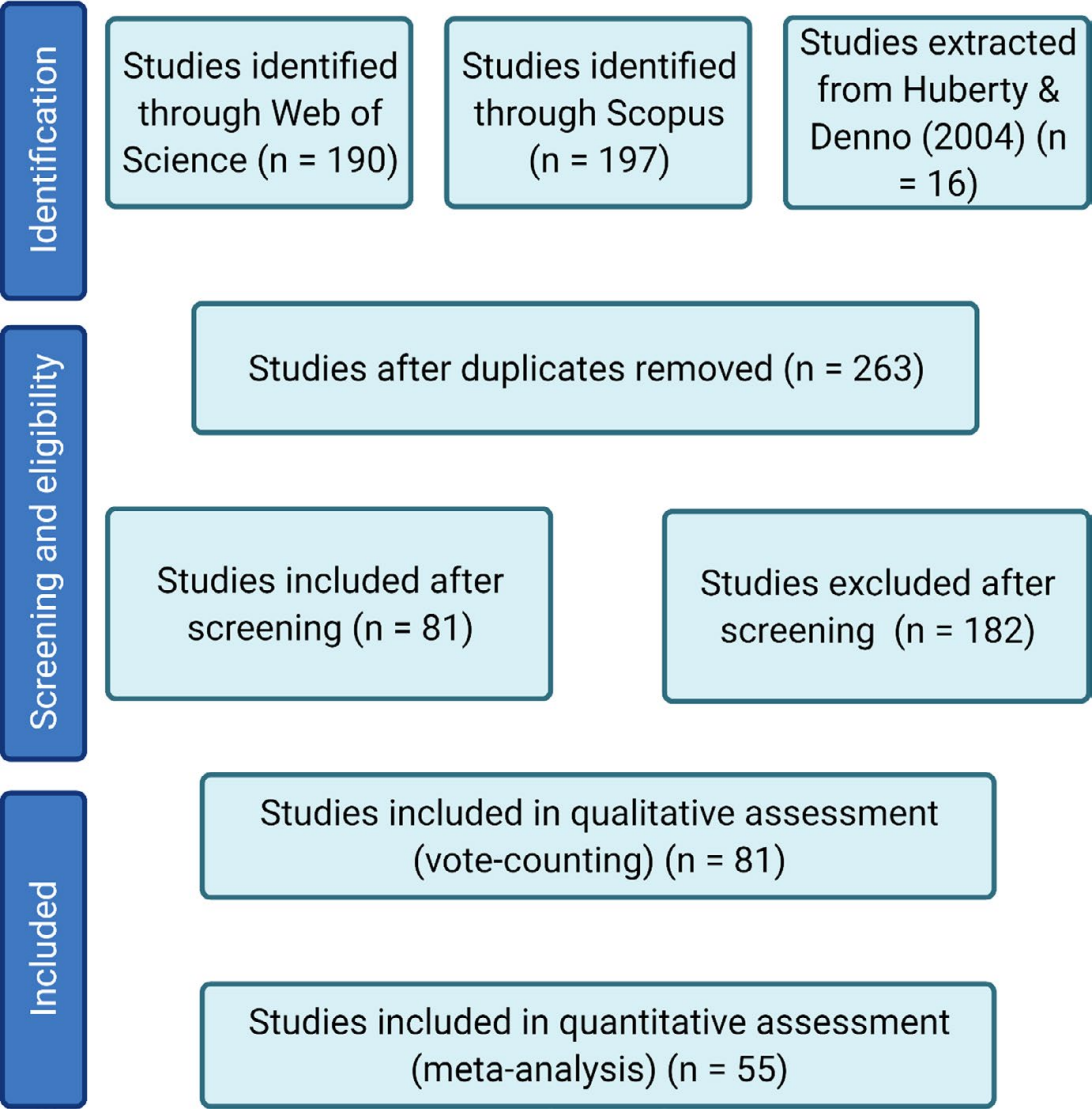
PRISMA diagram

The search terms “Drought” AND “Aphid” were used to conduct a literature search of both the Web of Science and Scopus databases (with a publication cutoff date of September 2020), and two database searches were included to maximize the number of studies included (the overlap between Web of Science and Scopus is only 40%–50%: Nakagawa et al., 2017). 190 papers were identified from the Web of Science database and 197 from the Scopus database. After removing duplicates, 247 published papers were extracted. A previous meta‐analysis which examined insect responses to drought (Huberty & Denno, 2004) was screened, and an additional 16 studies were identified. This produced a pool of 263 studies published between 1958 and 2020. See Figure 1 for the PRISMA flow diagram, constructed following Moher et al. (2009). To be considered for inclusion in the analysis, papers had to satisfy the following criteria: (a) to be primary literature presenting data on the responses of at least one aphid species to drought relative to an unstressed control treatment, (b) report aphid responses as the effect of drought on a measure of aphid fitness, and (c) present the responses so that an estimation of the treatment differences could be determined alongside an estimate of the variation. A total of 55 studies satisfied these criteria. A further 26 studies reported data for aphid fitness but did not display the data; these studies were excluded from the meta‐analysis but included in qualitative “vote‐counting” assessment. The full range of studies are detailed in Appendix S1 and Appendix S2.

Extracted data covered a worldwide distribution, with results reported for every continent apart from Antarctica. Various experimental designs were used, the majority of studies used controlled environment rooms and an equal number of studies collected data under field (including polytunnel and field trials) or glasshouse conditions. Appendix S3 shows the number of studies across six geographical regions (Africa, Asia, Australasia, Europe, North America, and South America) as well as the number of studies per experimental condition (controlled environment, field or polytunnel, glasshouse).

Heterogeneity is routinely expected and accepted in meta‐analyses (Higgins, 2008). Acceptance of heterogeneous data is dependent on whether the inclusion criteria are sound and the underlying data correct (Higgins, 2008). Extracted data were assessed for heterogeneity by measuring Cochran's *Q* (Cochran, 1954).

#### 2.1.2 Data extraction, pooling, and grouping

Aphid fitness data were extracted from drought and control (unstressed) treatments. Where reported, the mean value and an indication of the variation around the mean were extracted using WebPlotDigitizer v.4.2 (Rohatgi, 2020. Weblink: https://automeris.io/WebPlotDigitizer). Where median and interquartile ranges were reported, means and standard deviation were estimated following Luo et al. (2018) and Wan et al. (2014). Data were extracted for the following aphid fitness parameters: fecundity (measures of daily, lifetime, and mean fecundity and life‐history parameters related to reproduction, such as the intrinsic rate of increase), population size or aphid density/abundance, aphid development (time until adulthood and time until first reproduction), aphid biomass, or aphid lifespan. The effect size (Hedges' *g*; Cooper et al., 2019) was calculated from aphid responses under drought relative to aphid responses under control conditions. Hedges' *g* was selected as this parameter performs better across studies with a lower sample size, when compared with Cohen's D or Glass's Delta. Where multiple drought treatments were imposed, data were extracted from the control and the most severe drought treatment.

Where multiple formats of a fitness parameter were reported (e.g., fecundity reported in terms of mean fecundity and lifetime fecundity), data were pooled across different measures of the core parameter to provide one response per parameter assessed. Data were further pooled across any other experimental treatments imposed in the study (e.g., in Xing et al. (2003) drought and control conditions were pooled across three CO_2_ treatments), and within aphid species to provide one data point per aphid species per fitness parameter. Data were collated separately for each aphid species—host plant species combination. This pooling method produced 93 unique data points over the 55 studies.

Two datasets were compiled based on these data. In the “global” dataset, data were pooled within each study across fitness parameters, plant hosts, and aphid species to produce 55 data points, that is, one pooled effect size per study, reporting the overall aphid responses to drought. Within the “global” dataset, the calculated hedges' *g* values for developmental fitness parameters were inverted from positive to negative values to align with the net direction of other fitness parameters (this was required because a positive hedges' *g* value for development represents a fitness decrease, compared with the other parameters for which a fitness decrease would result in a negative hedges' *g* value). In the “expanded” dataset, effect sizes were calculated separately for each fitness parameter and aphid–host plant combination in the study. To ensure that the direct comparison of different aphid fitness parameters was justified for inclusion in the analysis, the calculated hedges' *g* values for each data point were plotted (Appendix S4) to confirm that data were evenly distributed and not clustered into categories.

Extracted data contained information on 23 aphid species (Table 1). To facilitate analysis of biological factors that might influence aphid responses to drought, the data were categorized into three groups: a “Tribe” grouping based on the taxonomic tribe of the aphid species; an “Aphid Host Range” category based on whether the aphid species is a specialist or generalist feeder; and a “Plant Family” category based on the family of the host plant. Specialist aphids were defined as aphid species whose secondary hosts consist of only one plant family (e.g., cereal aphids such as *Rhopalosiphum padi* where the secondary host plants consist only of members of the Poaceae), whereas aphids were classed as generalists if their secondary hosts included plant species across two or more plant families (Schoonhoven et al., 2005). A geographical group, based on the region the study was from, was also created to enable analysis across geographical regions.

**TABLE 1:** Information on the aphid species included in the meta‐analysis, the agricultural and ecological importance of each species, and the number of data points present in the “expanded” dataset

Aphid species	Common name	Aphid tribe/Specialism (generalist or specialist)	Agricultural and ecological importance	Number of data points: “expanded” dataset and study from which data were extracted^a^
*Adelges abietis* Linnaeus	Eastern spruce gall aphid	Adelgini (Other)/Specialist	Common pest of Norway and Sitka spruce. Stem‐mother forms pineapple‐shaped gall.	2 datapoints from studies 10 and 11
*Aphis glycines* Matsumura	Soybean aphid	Aphidini/Specialist	Significant pest of soybean across North America and Asia	1 datapoint from study 31
*A. pomi* DeGeer	Apple aphid	Aphidini/Specialist	Significant pest of apple trees, specifically on nursery stock. Colonies can be attended by ants	1 datapoint from study 29
*Acyrthosiphon pisum* (Harris)	Pea aphid	Macrosiphini/Generalist	Widespread in temperate regions. Serious pest of the Fabaceae. Vector of over 30 plant viruses	4 datapoints from studies 7, 20, 26, 43
*Brevicoryne brassicae* (Linnaeus)	Cabbage aphid	Macrosiphini/Generalist	Wax‐covered aphid. Widely distributed throughout Europe, major pest of Brassicaceae	8 datapoints from studies 23, 37, 47, 48. 49,
*Cinara costata* (Zetterstedt)	Mealy spruce aphid	Eulachnini (Other)/Specialist	Wax‐covered aphid. Can cause damage to spruce trees. Unlike other *Cinara spp. C. costata* is not readily attended by ants.	1 datapoint from study 24
*C. pinea* (Mordvilko)	Large pine aphid	Eulachnini (Other)/Specialist	Forestry pest of Scotts pine and other *Pinus spp*. Often found on young pine trees.	1 datapoint from 32
*Chaitophorus leucomelas* Koch	Black poplar leaf aphid	Chaitophorini (Other)/Specialist	A widely distributed pest of poplar. Colonies can reside in the galls formed by other insects and can occasionally be attended by ants.	1 datapoint from study 39
*Diuraphis noxia* (Mordvilko)	Russian wheat aphid	Macrosiphini/Specialist	A significant cereal pest in South Africa and North America. Vector of barley yellow dwarf virus.	3 datapoints from studies 2, 16, 34
*Dysaphis plantaginea* (Passerini)	Rosy apple aphid	Macrosiphini/Specialist	Widely distributed across temperate regions and a significant pest of apples. Colonies develop in galls and are attended by ants.	1 datapoint from study 41
*Elatobium abietinum* (Walker)	Green spruce aphid	Macrosiphini/Specialist	Distributed across Europe and North America. Can cause economic and environmental damage by causing needle defoliation on Spruce trees.	6 datapoints from studies 2, 4, 5, 6
*Lipaphis erysimi* (Kaltenbach)	Wild crucifer aphid	Macrosiphini/Specialist	Can feed on various Brassicaceae crops.	1 datapoint form study 23
*Macrosiphum euphorbiae* (Thomas)	Potato aphid	Macrosiphini/Generalist	A significant pest species with worldwide distribution Can cause significant economic damage through the transmission of over 20 plant viruses.	7 datapoints from studies 8, 33, 40
*Metopolophium festucae* subsp. *cerealium* (Theobald)	Fescue aphid	Macrosiphini/Specialist	A subspecies of *M. festucae* which can be a significant pest of cereals and grasses.	2 datapoints form study 18
*Myzus persicae* (Sulzer)	Peach‐potato aphid	Macrosiphini/Generalist	Significant pest with worldwide distribution. Broad host range. Can cause significant economic damage through the transmission of over 40 plant viruses.	12 datapoints from studies 27, 35, 38, 44, 47, 48, 49, 50
*Pemphigus betae* Doane	Sugarbeet root aphid	Hormaphidini (Other)/Specialist	Root‐feeding aphid, a main economic pest of sugar beet.	2 datapoints from study 30
*Phloeomyzus passerinii* (Signoret)	Poplar woolly aphid	Phloeomyzinae (Other)/Specialist	Distributed in temperate regions. Significant economic pest of poplar.	1 datapoint from study 14
*Rhopalosiphum maidis* (Fitch)	Green corn aphid	Aphidini/Specialist	Widely distributed worldwide. A significant pest of cereals. Commonly attended by ants. Vector of several plant viruses.	2 datapoints from studies 34, 42
*R. padi* (Linnaeus)	Bird cherry‐oat aphid	Aphidini/Specialist	Widely distributed worldwide, a significant pest of cereals and a vector of numerous plant viruses. Populations on the primary host can be attended by ants.	18 datapoints from studies 3, 9, 15, 18, 19, 21, 28 53, 55
*Sitobion avenae* Fabricius	Wheat aphid	Macrosiphini/Specialist	Widespread pest of cereals with worldwide distribution. A vector of several economically important plant viruses.	16 datapoints from 1, 13, 17, 25, 36, 45, 46, 51, 54
*Sipha maydis* Passerini	Bristly black grass aphid	Siphini (Other)/Specialist	Widely distributed pest that feeds on numerous Poaceae species, including cultivated crops and perennial grasses. Can be attended by ants.	1 datapoint from 28
*Schizaphis graminum* (Rondani)	Spring green aphid	Aphidini/Specialist	Widely distributed pest of cereals. Can vector a range of plant viruses.	1 datapoint from study 12
*Therioaphis trifolii* (*f. maculate*) (Buckton)	Spotted alfalfa aphid	Panaphidina (Other)/Specialist	Widespread pest of Fabaceae.	1 datapoint from study 7

Note

Information included in this table was primarily extracted from Centre for Agriculture and Bioscience International (CABI) databases.

^a^ Full study references are contained in Appendix S2.

#### 2.1.3 Screening of studies reporting plant responses to drought

Studies were screened for inclusion in an additional meta‐analysis to determine the impact of drought on the host plant. To be considered for inclusion, studies had to satisfy the following criteria: (a) present data on the responses of at least one plant species to drought relative to a controlled condition; (b) report responses as the effect of drought on either a measure of vigor (including mass, height, and growth), tissue nitrogen (N) or amino acid concentration, or plant chemical defense (e.g., secondary metabolite or phytohormone concentration); and (c) report an estimation of the differences alongside the variation. From the pool of 55 studies, 32 reported effects of drought on plant vigor (i.e., dry matter accumulation, plant growth, and leaf/tiller production), 12 reported a measure of tissue N or amino acid concentration, and ten reported tissue defensive compound concentrations. The effect size (Hedges' *g*) was calculated as described above. Data were pooled at the study level into measures of vigor, N or amino acid concentration, and defensive compound concentrations, resulting in three plant sub‐datasets: vigor, nutritional, and defensive.

### 2.2 Data analysis

#### 2.2.1 Vote‐counting procedure

For the qualitative vote‐counting analysis, studies were screened for whether a significant effect of drought on aphid fitness was detected, and whether the direction of the effect was positive or negative. Studies which reported nonsignificant results were categorized as null response. Data were deemed as significant based on the statistical reporting in each study (significance was determined by *p* = <0.05). For studies measuring the responses of several aphid species (such as Foote et al., 2017), the results of the statistical analysis at the drought treatment level, not the species × drought interaction level, were used to determine whether the observation was significant among all aphid species.

#### 2.2.2 Statistical analysis

Statistical analysis was carried out using R version 4.0.3, with additional packages ggplot2 v.3.3.2 (Wickham, 2016), meta v.4.15‐1 (Balduzzi et al., 2019), and metafor v.2.4‐0 (Viechtbauer, 2010). A total of 81 and 55 studies were included in the vote counting and meta‐analysis, respectively (detailed in Appendix S1 and Appendix S2).

#### 2.2.3 Aphid meta‐analysis

Two main meta‐analyses were carried out using the two datasets described above: (a) an analysis using the “global” dataset pooled across aphid species and host plant giving one pooled effect size per study (55 data points) and (b) an analysis using the “expanded” dataset which was pooled at the aphid species and host plant levels and separated by aphid fitness parameter, giving multiple pooled effect sizes per study (93 data points). For each meta‐analysis, random‐effects meta‐analysis models fitted with restricted maximum‐likelihood distribution were used. Study number was included as a random effect in each model, and all models were weighted using an inverse‐variance weighting method to account for within‐study variation and between‐study variation.

For the first dataset (“global”), an initial analysis was carried out to examine the broad effect of drought stress on aphid fitness. Following this, subgroup analysis (Borenstein & Higgins, 2013) was used to identify any differences in aphid responses to drought that were caused by either aphid biology (i.e., differences between aphid tribes), geographical region (continent), or by host–plant biology (plant family). Briefly, the subgroup analysis involved building three additional models, an aphid tribe, geographical region, and plant family model, respectively, that had one of these factors included as model moderators; moderator testing (Wald‐type test) was carried out to identify differences between the categories. As studies used various methodologies to implement drought, these methods were allocated into one of five categories: FC (studies where % reduction in field capacity was used); DI (studies where decreased volume of irrigation was used); GM (studies which used a gravimetric method to adjust irrigation); CC (studies which used a calibration curve to help advise water irrigation regimes); and RW (studies where irrigation was simply restricted or withheld from the drought treated plants). The effect of these drought treatments on aphid fitness was examined to confirm that different methodologies used to initiate drought did not vary in their effects (Appendix S5).

In the second dataset (“expanded”), data were analyzed in a similar way to the method above: briefly, a random linear mixed effects model fitted with restricted maximum‐likelihood distribution with study included as a random term was used to examine aphid responses to drought stress. Following this, two subgroup analyses were carried out in order to identify any differences in aphid responses between generalist and specialist aphids and to investigate how drought affects different aphid fitness parameters.

#### 2.2.4 Plant meta‐analysis

32, 12, and ten studies also reported the effect of drought on plant vigor, plant tissue nutrient concentration, and tissue defensive compound concentrations, respectively. An individual model was used to analyze each plant response. Each plant response was examined using a random linear mixed effects model fitted with restricted maximum‐likelihood distribution with study included in each model as a random effect. Models were weighted using an inverse‐variance weighting method to account for within‐study variation and between‐study variation.

#### 2.2.5 Measuring publication bias

For all main meta‐analysis models, funnel plots were created to test for publication bias and a rank correlation test was carried out to test for funnel plot asymmetry (Appendix S6). Additionally, as most null results go unpublished, failsafe analysis using Orwin's method (Orwin, 1983) was employed to estimate the number of studies reporting a null effect that would be required to reduce the observed average effect size to −0.1. This analysis estimated *n* = 261 and *n* = 2,228 null studies required to reduce the observed average effect size to −0.1 in the aphid and plant datasets, respectively. Appendix S7 shows the forest plot for each of the main meta‐analysis datasets.

## 3 RESULTS

### 3.1 Aphid fitness is reduced under drought

**FIGURE 2:**
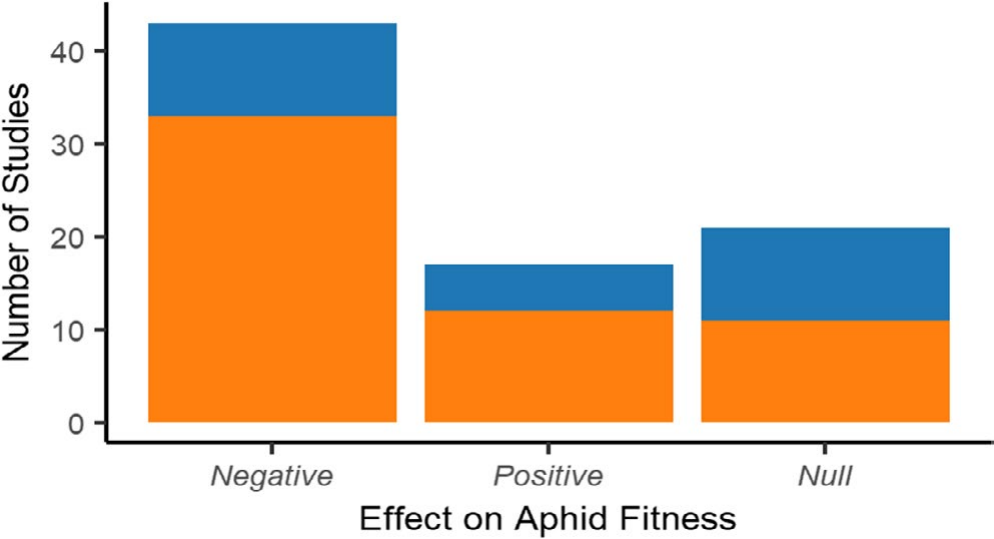
The number of studies reporting negative, positive, or null effects of drought stress on aphid fitness. Bars are colored to indicate the proportion of studies in each category that were included in the full meta‐analysis (Orange) versus those in the complete dataset (Blue)

The vote‐counting procedure (Figure 2) indicated that aphid fitness is reduced when exposed to drought‐stressed plants. Quantitative analysis of the pooled data through meta‐analysis also showed that aphid fitness is generally reduced under drought conditions (Hedges' *g* = −0.57; *n* = 55; *df* = 54; *p* = <0.001). Pooled data were significantly heterogeneous, indicated by Cochran's *Q* (*Q*_E_ = 404.34; *df* = 54; *p* = <0.001), signifying that random‐effects meta‐analysis methods represent the best approach for data analysis. No trends over publication time were observed for pooled aphid responses (Appendix S8).

**FIGURE 3:**
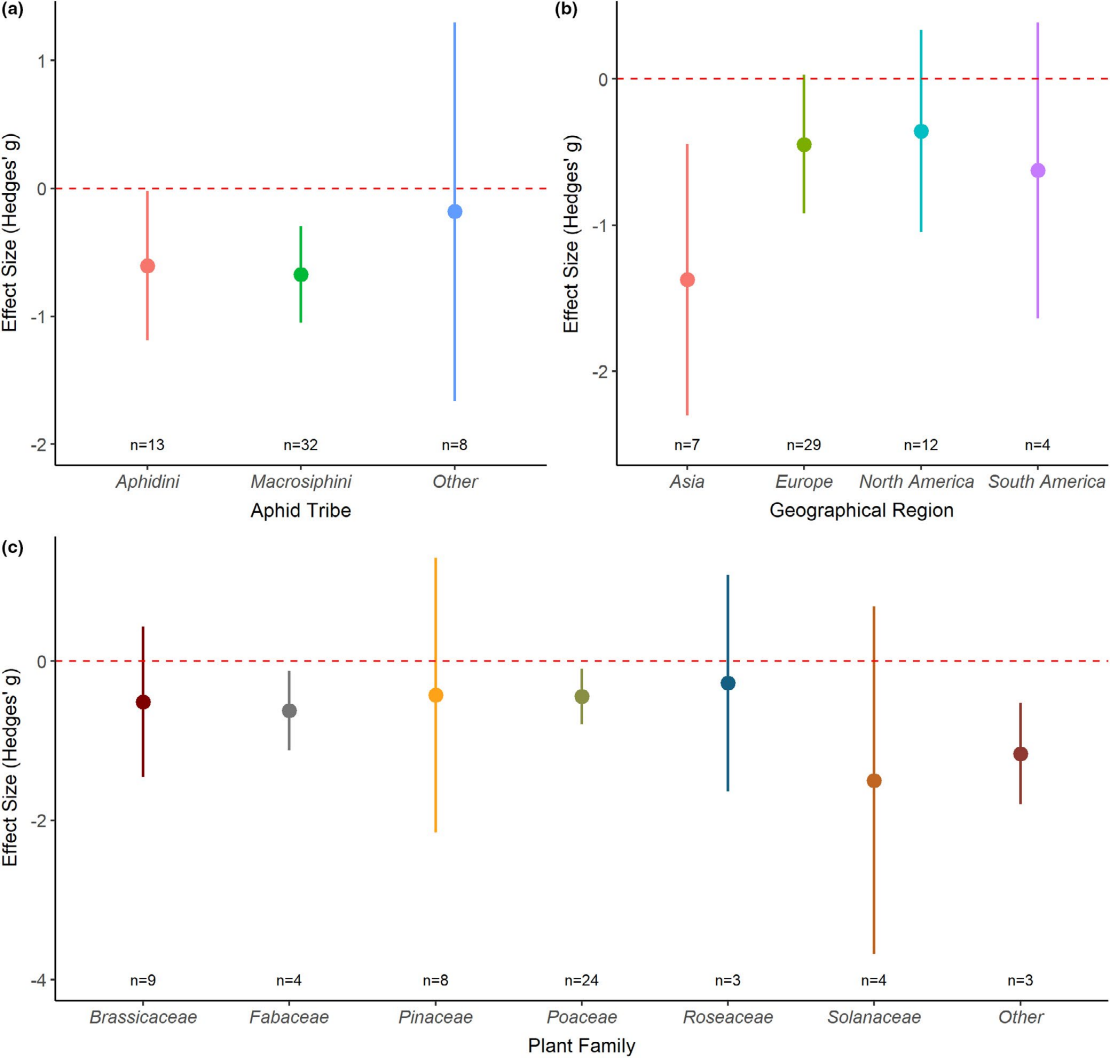
Aphid responses to drought stress conditions across different aphid tribes (a), Geographical Regions (b), and different plant families (c). Data use the “global” dataset where response variables were pooled to produce one effect size per study. Graph displays the mean effect size (Hedges' *g*) and the 95% confidence intervals for the different plant‐aphid systems identified from the extracted data. Red dashed line represents zero effect size

Examination of whether biological or geographical factors might influence aphid responses to drought stress was carried out by including aphid tribe, geographical region, and host plant family as model moderators during subgroup analysis. There was a clear negative effect of drought on aphid fitness for studies carried out in Asia and for aphids feeding on members of the Poaceae or Fabaceae family, and although other groups showed a general negative trend in response to drought stress, the significance of this effect varied. Subgroup analysis indicated that there were no significant differences among the different aphid tribes examined (test of moderators: *Q*_M_ = 0.37; *df* = 3; *p* = .946), over the different geographical regions (test of moderators: *Q*_M_ = 5.20; *df* = 3; *p* = .157), or across different host plant families (test of moderators: *Q*_M_ = 11.74; *df* = 6; *p* = .068), indicating that drought stress has a similar effect on aphid fitness across multiple plant–aphid systems and across the geographical regions (Figure 3). However, due to low replication for some groups, a comprehensive comparative analysis was not possible (plant families with *n* = <3 were combined into “Other” and low levels of replication, *n* = <3, from Africa and Australasia meant these regions could not be included in the geographic region analysis).

**FIGURE 4:**
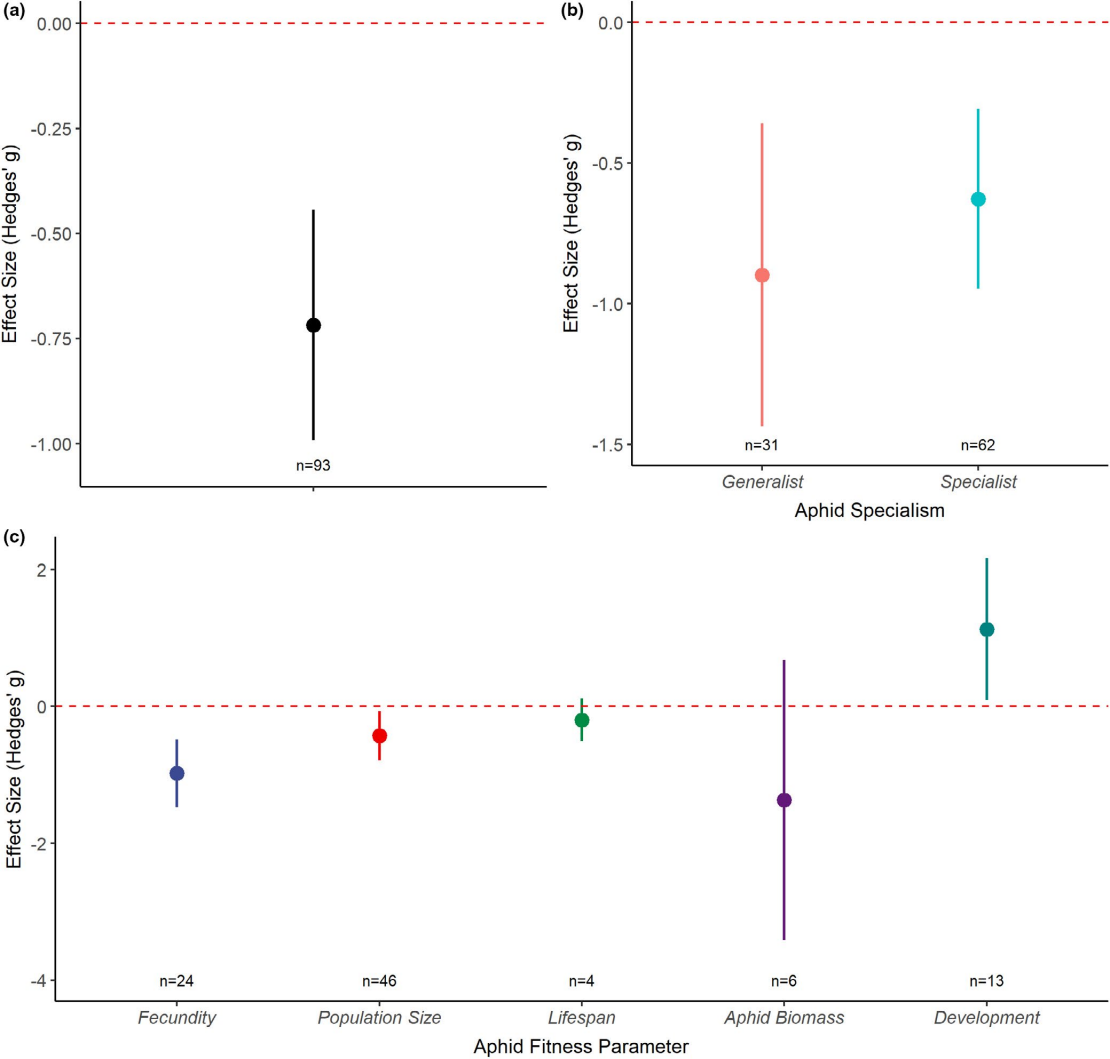
Responses of aphids to drought stress using the “expanded” dataset which included an effect size per response variable and for each aphid species measured in each study. Graphs display the mean effect size (Hedges' *g*) and the 95% confidence intervals. (a) Shows the mean effect size for all the combined data included in the “expanded” dataset, (b) shows the effect size for generalist and specialist aphids, (c) shows the mean effect size for the various aphid fitness parameters reported. Red dashed line represents zero effect size

Subsequent analysis used the “expanded” dataset. The “expanded” dataset comprised 93 data observations over the 55 studies. Meta‐analysis of all datapoints included in this “expanded” dataset indicated that aphid fitness was, generally, reduced under drought (Hedges' *g* = −0.72; *n* = 93; *df* = 92; *p* = .003; Figure 4a). This dataset was used to explore potential differences between aphid specialism (specialist vs. generalist aphids) and to determine whether drought has contrasting effects on aphid fitness parameters. Subgroup analysis of aphid specialism indicated that there was no significant difference in the response of aphids to drought stress between specialist and generalist aphids, with drought stress having a similar negative effect on both (test of moderators: *Q*_M_ = 0.04; *df* = 1; *p* = .835; Figure 4b). Most aphid fitness parameters examined, especially parameters intrinsically associated with aphid abundance (fecundity and population size) and development, were negatively affected by drought (Figure 4c). Subgroup analysis indicated that there was no significant difference between fitness parameters (test of moderators: *Q*_M_ = 6.39; *df* = 4; *p* = .172). It should be noted that for the above analyses, the observed effect size for measures of aphid development was inversed; as explained in the Materials and Methods section, this was done because a positive effect on aphid development equates to a negative fitness consequence for aphids; therefore, the inverted value was used in order to align this with the direction of other fitness consequences during statistical analysis.

### 3.2 Drought‐stressed plants show reduced vigor and increased tissue concentrations of defensive compounds

**FIGURE 5:**
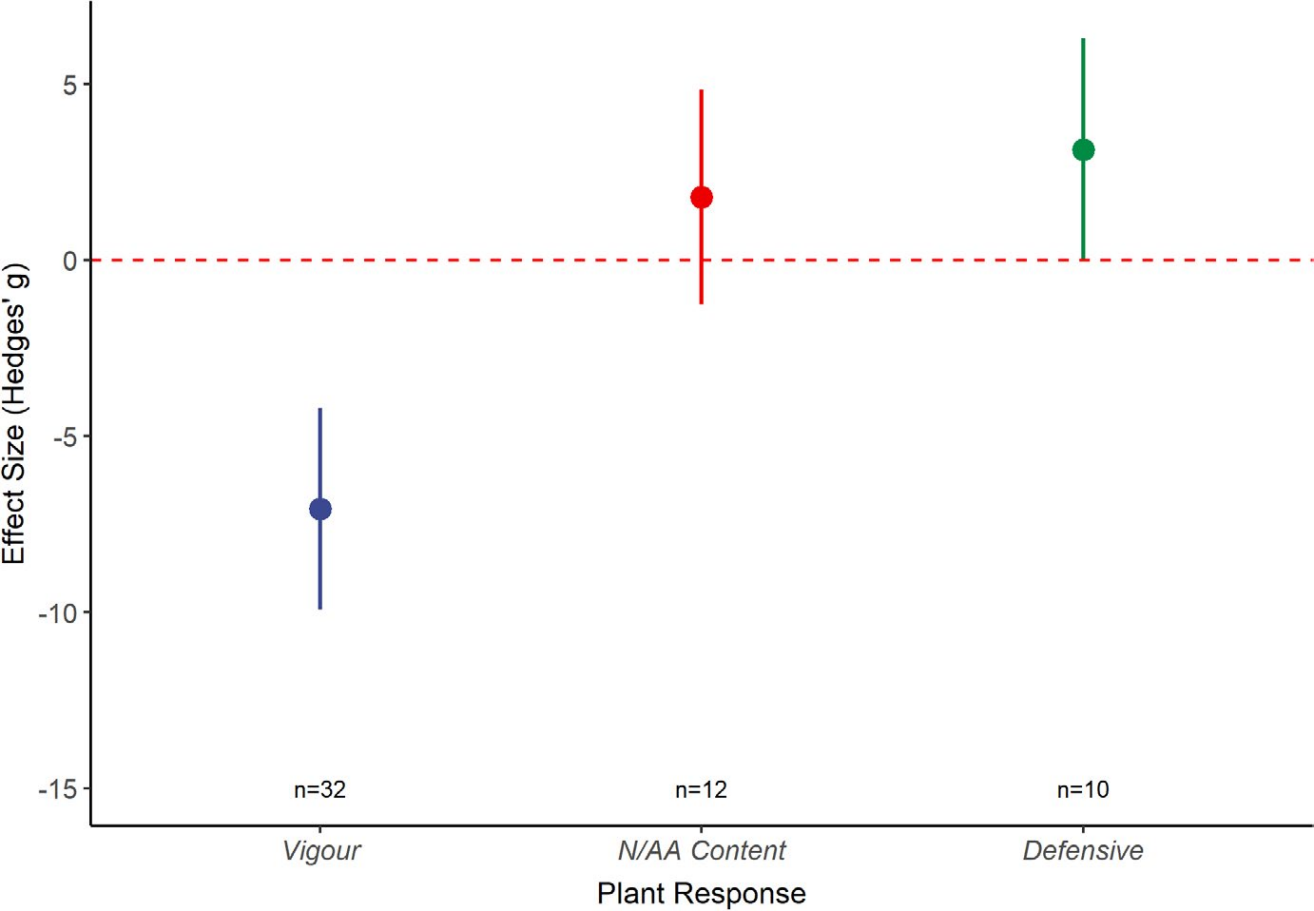
The effect of drought stress on plant vigor using the “plant” dataset where measures of plant vigor (vigor), N or amino acid (AA) concentration (N/AA Content), or tissue concentrations of defensive compounds (Defensive) were reported. Graph displays the mean effect size (Hedges' *g*) and the 95% confidence intervals for the different plant–aphid systems identified from the extracted data. Red dashed line represents zero effect size

Meta‐analysis of the pooled plant responses to drought indicated that exposure to drought has an overall negative effect on plant vigor (Hedges' *g* = −7.06; *n* = 32; *df* = 1; *p* = <0.001; Figure 5) and, on average, results in more chemically defended plant tissues (Hedges' *g* = 3.14; *n* = 10; *df* = 1; *p* = <0.001; Figure 5). However, tissue N and amino acid concentrations did not increase consistently (Hedges' *g* = 1.79; *n* = 12; *df* = 1; *p* = .206; Figure 5). No trends over publication time were observed for any plant responses (Appendix S8). The consistency of the relation between drought effects on aphid fitness, plant vigor, and plant chemical defense, but not plant nutritional quality, is shown in Appendix S9.

## 4 DISCUSSION

This study provides the first comprehensive assessment of aphid and host plant responses to drought stress, analyzed in terms of aphid fitness and plant vigor, nutritional quality, and chemical defense. The meta‐analysis supported our prediction that drought reduces aphid fitness, and this effect was linked most consistently with reduced plant vigor rather than altered tissue nutrient concentrations, although increased chemical defense of plant tissues might also play a role. Our study includes data extracted from 81 (qualitative assessment) and 55 (quantitative assessment) studies, including 42 papers for quantitative assessment published between 2003 and 2020 that were not included in an earlier meta‐analysis across different insect feeding guilds (Huberty & Denno, 2004). Our findings, therefore, represent a significant advance in knowledge on the effects of drought on sap‐feeding insects with a novel focus of drought effects on aphids as an ecologically important insect group.

### 4.1 Plant vigor could explain drought effects on aphid fitness

The primary finding that aphid fitness is reduced when feeding on drought‐stressed hosts confirms the more general findings of Huberty and Denno (2004) for sap‐feeding insects. Our study goes further, however, by providing evidence for the underlying mechanisms. We show that decreased aphid fitness is most frequently associated with the negative effects of drought on plant growth (vigor), with some evidence that plant tissue concentrations of defensive chemicals or signaling compounds play an important role. However, we found no effect of drought stress on the plant tissue nitrogen or amino acid concentrations. This null effect of drought on tissue nitrogen physiology could be a consequence of aphid presence, as some aphid species are able to remobilize plant nutrients (Sandström et al., 2000), or experimental design of the studies included in the analysis, as drought methodology and length of exposure to drought stress can influence tissue nitrogen concentration (He & Dijkstra, 2014). It should be noted that, out of the studies included in our meta‐analysis, over half measured plant vigor (32 studies) while only around one‐fifth measured tissue concentrations of defensive compounds (*n* = 10) or tissue nitrogen or amino acid concentration (*n* = 12). Most of these studies showed a strong association between decreased aphid performance, reduced plant vigor, and increased tissue concentrations of plant‐defensive compounds. This finding is consistent with our prediction and is supported by a large body of literature reporting elevated chemical defense and decreased plant growth and vigor under drought (Beetge & Krüger, 2019; Templer et al., 2017; Xie et al., 2020). As less vigorous plants have a higher concentration of biochemical defenses, drought stress could lead to increased aphid exposure to plant‐defensive compounds, leading to reduced fitness due to feeding deterrence and decreased phloem ingestion.

A secondary aim of this meta‐analysis was to examine the consistency of drought effects across aphid groups and aphid–plant systems to identify factors which could explain differential effects (Hale et al., 2003; Oswald & Brewer, 1997). All aphid tribes assessed showed decreased aphid fitness in response to drought, and a similar response was observed when aphids were categorized as specialists or generalists. However, there was an overrepresentation of the Aphidini and Macrosiphini tribes (45 out of the 55 studies) because many empirical studies focus on agriculturally or ecologically important aphid species, which are widely represented in these two tribes (Choi et al., 2018; Kim & Lee, 2008). This limits the extent to which differences between tribes can be detected and further experimental work would be needed to confirm consistent drought responses across aphid groups. For example, it could be hypothesized that aphid species which actively remobilize plant nutrients (e.g., *D. noxia*; see Sandström et al., 2000) are less affected by drought than species that are unable to maintain a sufficient supply of plant nutrients. Similarly, species that can sequester plant‐defensive chemicals (e.g., *Brevicoryne brassicae* (Linnaeus); see, Kazana et al., 2007) might better tolerate increased concentrations of toxic plant chemicals in response to drought. Our assessment of the responses of aphids on different plant families indicated that aphids on drought‐stressed Poaceae and Fabaceae were more susceptible to drought than aphids feeding on other plant families. Although aphid responses on the other plant groups also showed an overall negative response to drought stress, including groups with high levels of chemical defenses (i.e., Brassicaceae), these were more variable. Again, the level of replication limits the extent to which we can compare aphid responses on different plant types. Nonetheless, these findings confirm that the effect of drought on herbivorous insects is primarily mediated by general changes in plant physiology, as indicated by Cornelissen et al. (2008).

### 4.2 The role of host plant defense in aphid responses to drought

Although based on a relatively small number of studies, our analysis indicated that plant chemical defense is elevated under drought, and this correlates with reduced aphid fitness. Several studies highlight the effects of antiherbivore plant resistance strategies on aphid fitness under benign conditions (Greenslade et al., 2016; Guerrieri & Digilio, 2008), but few studies have examined whether host plant resistance against aphids is modified under climate stress. From the 55 studies assessed here, only five included observations of aphid responses on both susceptible‐ and (partially)‐resistant plant types (Björkman, 2000; Dardeau et al., 2015; Guo et al., 2016; Oswald & Brewer, 1997; Verdugo et al., 2015) with one further study examining aphid responses on resistant plants only (Ramirez & Verdugo, 2009). Additionally, three studies compared aphid responses on drought tolerant and drought susceptible host plants (Farias et al., 1995; Quandahor et al., 2019; Rousselin et al., 2018) and only one study examined the interactive effects of aphid resistance and drought tolerance (Grettenberger & Tooker, 2016). Such a low level of representation means that the interactive effect between plant resistance and drought could not be investigated empirically using meta‐analysis. Of these studies, four report similar findings: Aphid fitness is reduced on both susceptible and resistant plant hosts (Björkman, 2000; Dardeau et al., 2015; Guo et al., 2016; Oswald & Brewer, 1997), with a smaller reduction in fitness on the resistant host plant than on the susceptible host plant (Dardeau et al., 2015; Oswald & Brewer, 1997). These findings indicate that while aphid fitness is reduced on resistant plants under benign conditions, under drought aphid fitness decreases to similarly low values on susceptible and resistant plants. This highlights a significant knowledge gap in our understanding of how plant resistance traits are affected by environmental stress, which is becoming increasingly important for successful pest management in changing climatic conditions.

**FIGURE 6:**
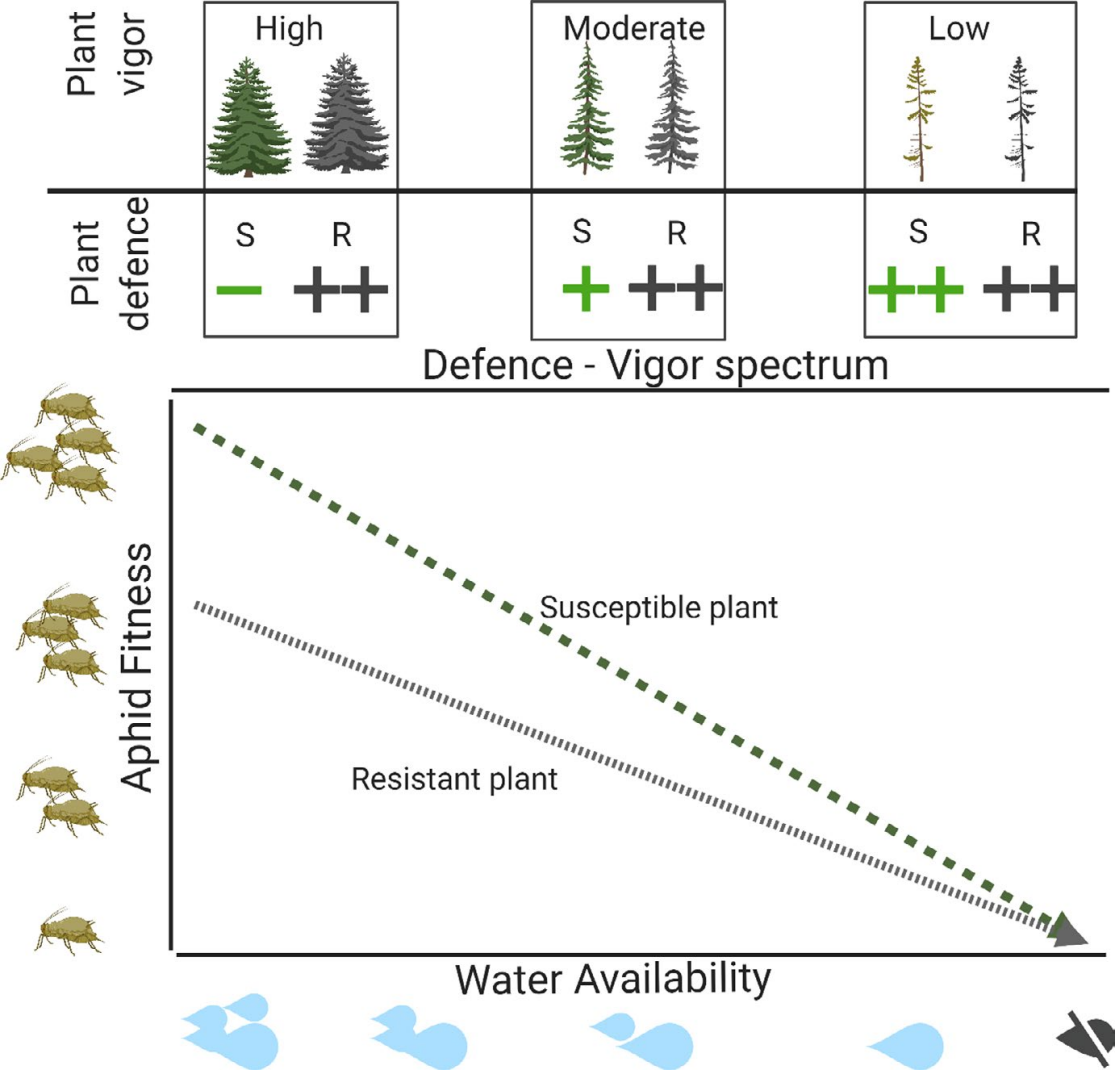
Conceptual representation of the plant resistance hypothesis (PRH). As water availability decreases, plant defense increases and plant vigor declines, leading to reduced aphid fitness. Basal levels of aphid fitness under conditions of ample water availability differ between the susceptible (S, green line) and the resistant (R, gray line) plant types. Under drought, aphid fitness is reduced on both plant types; however, the extent of this reduction is greater for the susceptible plant (high to low fitness) than the resistant plant (intermediate to low fitness). This image made in BioRender © ‐ biorender.com

To stimulate further research into this potentially important interaction between plant resistance, herbivore success, and climatic conditions, we propose a simplified conceptual model to predict how aphids will respond to drought in relation to altered host–plant resistance, termed the plant resistance hypothesis (Figure 6). This expands upon previous conceptual models, the plant stress hypothesis and plant vigor hypothesis, which do not consider the potential differences in plant susceptibility to herbivorous insect pests. This new model suggests that under benign conditions, the basal level of aphid fitness will differ between the susceptible (high aphid fitness) and resistant (moderate‐to‐low aphid fitness) plant types, as will the level of plant defense: susceptible (low level of defense) versus resistant (high level of defense). Drought is likely to cause a reduction in plant vigor and decreased plant palatability for the aphid, characterized by elevated concentrations of plant‐defensive compounds (Inbar et al., 2001; Ozturk et al., 2002), leading to differential changes in the chemical defense of susceptible (from low to high concentration) and resistant (continually high concentration) plants. Differential levels of basal aphid fitness between the two plant types lead to differences in the extent to which aphid fitness is affected by drought depending on whether the host is a susceptible (from high fitness to low fitness) or resistant (from intermediate fitness to low fitness) plant type.

### 4.3 Drought‐induced reduction in aphid fitness could destabilize aphid‐trophic interactions

Aphids represent an important group of herbivorous insects from both an economic perspective, in relation to agricultural crop protection, and an ecological perspective, regarding the diverse community of higher trophic groups they support. The central finding of our meta‐analysis is that exposure to drought‐stressed hosts is detrimental to aphid fitness, although the extent of this effect is likely affected by host plant suitability as an aphid food source. Consequently, higher incidences of drought will have a detrimental effect on the terrestrial trophic networks that are supported by aphids: Aphids are widespread in vegetation systems globally, are abundant consumers of primary production in many ecosystems, and provide a food source for many trophic groups (Gilbert, 2005; Messelink et al., 2012; Roubinet et al., 2018). Our findings, largely based on aphid species in the Aphidini and Macrosiphini tribes, indicate that individual‐ and population‐level measures of aphid fitness are affected negatively by drought, suggesting that drought will have cascading consequences for host plant consumption by aphids and the abundance of other trophic groups; although it should be noted that only around 25% of the studies included in the meta‐analysis were field or polytunnel studies, so further examination of drought–aphid interactions under field conditions is required to further elucidate these potential consequences. If these findings translate to other aphid species and tribes, they imply that drought could reduce the availability of aphid hosts for parasites and pathogens (Ahmed et al., 2012; Aslam et al., 2013; Nguyen et al., 2007) and decrease food availability for aphid predators (Wade et al., 2017). Similarly, many aphids are tended by ants for their honeydew secretions (Stadler et al., 2003). These ants provide protective services to plants by deterring herbivory by other insect pests (Offenberg et al., 2004; Rosumek et al., 2009). While reduced aphid fitness might decrease plant consumption, it could also compromise the protective services delivered by ants: Lower aphid abundance, or decreased honeydew quantity or quality, could decrease ant attendance (see Stadler et al., 2003), thereby exacerbating the detrimental effects of drought by increasing plant exposure to additional biotic stressors.

Ecological networks often exist in stable equilibria (Landi et al., 2018; McQuaid & Britton, 2015), with a change in the abundance of one species or functional group leading to perturbations in abundance and diversity across the network (McQuaid & Britton, 2015). A drought‐induced reduction in aphid fitness might decrease abundance, mass, or quality of aphids available to support other trophic levels, with potential to destabilize population equilibria; a recent modeling study illustrated that the destabilizing effects of drought on aphid–parasitoid interactions lead to altered insect population cycles (Preedy et al., 2020). A key finding of this meta‐analysis was that plant resistance to aphids may influence the extent to which aphids are negatively affected by drought. The negative consequences of plant resistance for aphids generally include decreased fecundity (Greenslade et al., 2016; Leybourne et al., 2019) which could reduce aphid abundance for aphid‐natural enemies. This could have further consequences for aphid/insect distributions in regions that are experiencing more frequent drought events, especially when host plant species are more drought‐sensitive. Indeed, although we did not detect a significant difference between geographic regions in aphid responses to drought stress, we did observe a strong negative effect of drought on aphids in Asia, with weaker effects for aphids from the other regions examined. One potential explanation for this could be that plants or/and aphids that are native to the tropical climate of Asia are more drought‐sensitive.

Analyzing the effects on aphid–natural enemy interactions of drought, and its interactions with other determinants of host suitability, is therefore an important avenue for future research to understand the impacts on the composition and function of ecological networks and species distributions (e.g., Rodríguez‐Castañeda, 2013). This will improve our understanding of how drought and plant suitability characteristics might contribute to recent reports of increased rates of species loss and population declines (Leather, 2018; Saunders et al., 2020). Our conceptual model of the anticipated effects of drought on aphid fitness in relation to plant resistance, plant vigor, and chemical defense provides a basis for stimulating future research on insect–plant interactions under a changing climate.

## CONFLICT OF INTEREST

None declared.

## AUTHOR CONTRIBUTIONS

**Daniel J. Leybourne:** Conceptualization (lead); data curation (lead); formal analysis (lead); methodology (lead); validation (lead); visualization (lead); writing–original draft (lead); writing–review and editing (lead). **Katharine F. Preedy:** Formal analysis (supporting); methodology (supporting); supervision (supporting); validation (supporting); visualization (supporting); writing–original draft (supporting). **Tracy A. Valentine:** Supervision (supporting); writing–original draft (supporting). **Jorunn I. B. Bos:** Conceptualization (supporting); funding acquisition (lead); project administration (lead); supervision (supporting); writing–original draft (supporting). **Alison Karley:** Conceptualization (supporting); funding acquisition (supporting); project administration (supporting); supervision (lead); writing–original draft (supporting); writing–review and editing (supporting).

## Supporting information

Supplementary Material

## Data Availability

The dataset is available in the Dryad data repository (https://doi.org/10.5061/dryad.jdfn2z3bn).
