# Supplementary material for: Drought has negative consequences on aphid fitness and plant vigor: Insights from a meta‐analysis
Source: Ecol Evol. 2021 Jul 28;11(17):11915–29. doi: 10.1002/ece3.7957 (PMC8427572; doi:10.1002/ece3.7957)

**Appendices**

**Appendix S1:** References for the 25 studies which were included in the “vote-counting” analysis but excluded from the full meta-analysis

1. Agele SO., Ofuya TI. & James PO. (2006). Effects of watering regimes on aphid infestation and performance of selected varieties of cowpea (*Vigna unguiculata* L. Walp) in a humid rainforest zone of Nigeria. *Crop Protection* **25**, 73-78.
2. Banfield-Zanin JA. & Leather SR. (2014). Frequency and intensity of drought stress alters the population size and dynamics of *Elatobium abietinum* on Sitka spruce. *Annals of Applied Biology* **165**, 260-269.
3. Björkman C. (2000). Interactive effects of host resistance and drought stress on the performance of a gall-making aphid living on Norway spruce. Oecologia **123**, 223-231
4. Braun S. & Flückiger W. (1984). Increased population of the aphid *Aphis pomi* at a motorway. Part 2-The effect of drought and deicing salt. *Environmental Pollution. Series A, Ecological and Biological,* **36,** 261-270
5. DeVries NEL. & Manglitz GR. (1982). Spotted alfalfa aphid (*Therioaphis maculata* (Buckton)) (Homoptera: Aphididae): water stress, amino acid content and plant resistance. *Journal of the Kansas Entomological Society* **55**, 57–64
6. Forbes AE., Harvey CT. & Tilmon KJ. (2005). Variation in the responses of spotted alfalfa aphids, *Therioaphis maculata* Buckton (Homoptera: Aphididae) and pea aphids, *Acythosiphon pisum* Harris (Homoptera: Aphididae) to drought conditions in alfalfa (*Medicago sativa* L., Fabaceae). *Journal of the Kansas Entomological Society*, **78,** 387-389
7. Gange AC. & Brown VK. (1989). Effects of root herbivory by an insect on a foliar-feeding species, mediated through changes in the host plant. *Oecologia,* **81**, 38-42.
8. Karley AJ., Emslie-Smith M. & Bennett AE. (2017). Potato aphid *Macrosiphum euphorbiae* performance is determined by aphid genotype and not mycorrhizal fungi or water availability. *Insect Science,* **24**, 1015 - 1024.
9. Kennedy JS., Lamb KP. & Booth CO. (1958). Responses of *Aphis fabae* Scop. to water shortage in host plants in pots. *Entomologia Experimentalis et Applicata,* **1**, 274-290.
10. Khan MAM., Ulrichs C. & Mewis I. (2010). Influence of water stress on the glucosinolate profile of *Brassica oleracea* var. italica and the performance of *Brevicoryne brassicae* and *Myzus persicae*. *Entomologia Experimentalis et Applicata,* **137**, 229-236.
11. Khan MAM., Ulrichs C. & Mewis I. (2011a). Drought stress - impact on glucosinolate profile and performance of phloem feeding cruciferous insects. In: Hale C, ed. Xxviii International Horticultural Congress on Science and Horticulture for People, 111-117.
12. Khan MAM., Ulrichs C. & Mewis I. (2011b). Effect of water stress and aphid herbivory on flavonoids in broccoli (*Brassica oleracea* var. italica Plenck). *Journal of Applied Botany and Food Quality-Angewandte Botanik,* **84**, 178-182.
13. Latimer JG & Oetting RD. (1994). Brushing reduces thrips and aphid populations on some greenhouse-grown vegetable transplants. *Hortscience,* **29**, 1279-1281.
14. McMurtry J. (1962). Resistance of alfalfa to spotted alfalfa aphid in relation to environmental factors. *Hilgardia* **32,** 501-539. DOI:10.3733/hilg.v32n12p501
15. Michels GJ Jr. & Undersander DJ. (1986). Temporal and spatial distribution of the Greenbug (Homoptera: Aphididae) on sorghum in relation to water stress. *Journal of Economic Entomology* **79**, 1221-1225.
16. Miles P., Aspinall D. & Rosenberg L. (1982). Performance of the cabbage aphid, *Brevicoryne brassicae* (L.), on water-stressed rape plants, in relation to changes in their chemical composition. *Australian Journal of Zoology*, **30**, 337-346.
17. Pons X. & Tatchell GM. (1995). Drought stress and cereal aphid performance. *Annals of Applied Biology,* **126**, 19-31.
18. Romo CM. & Tylianakis JM. (2013). Elevated temperature and drought interact to reduce parasitoid effectiveness in suppressing hosts. *PLoS ONE*, **8**.
19. Salas ML. & Corcuera LJ. (1991). Effect of environment on gramine content in barely leaves and susceptibility to the aphid *Schizaphis graminum*. *Phytochemistry,* **30**, 3237–3240.
20. Service PM. & Lenski RE. (1982). Aphid genotypes, plant phenotypes, and genetic diversity: a demographic analysis of experimental data. *Evolution,* **36**, 1276–1282
21. Teixeira N., Valim J., Oliveira M. & Campos W. (2020). Combining effects of soil silicon and drought stress on host plant chemical and ultrastructural quality for leaf-chewing and sap-sucking insects*. Journal of Agronomy and Crop Science*, **206** 187-201
22. Volpe V., Chitarra W., Cascone P., Volpe MG., Bartolini P., Moneti G., … Balestrini R. (2018). The association with two different arbuscular mycorrhizal fungi differently affects water stress tolerance in tomato. *Frontiers in Plant Science*, **9.**
23. Wearing CH. (1967). Studies on the relations of insect and host plant: ii. effects of water stress in host plants on the fecundity of *Myzus persicae* (Sulz.) and *Brevicoryne brassicae* (L.). *Nature,* **213**, 1052-1053.
24. Wearing CH. (1972). Responses of *Myzus persicae* and *Brevicoryne brassicae* to leaf age and water stress in brussels sprouts grown in pots. *Entomologia Experimentalis et Applicata,* **15**, 61-80.
25. Wearing CH. & Van Emden HF. (1967). Studies on the relations of insect and host plant: i. effects of water stress in host plants on infestation by *Aphis fabae* Scop, *Myzus persicae* (Sulz.) and *Brevicoryne brassicae* (L.). *Nature***, 213**, 1051-1052.

**Appendix S2:** References for the 55 studies which were included in the full meta-analysis. ǂ indicates which studies contained more than one data point when split into the “expanded responses” dataset. * Indicates which studies reported on plant physiological, defensive, or nutritional responses for inclusion in the plant meta-analysis

1. Ahmed SS., Liu D. & Simon J-C. (2017). Impact of water-deficit stress on tritrophic interactions in a wheat-aphid-parasitoid system. *PLoS ONE,* **12**, e0186599.
2. Archer TL., Bynum ED., Onken AB. & Wendt CW. (1995). Influence of water and nitrogen fertilizer on biology of the Russian wheat aphid (Homoptera: Aphididae) on wheat. *Crop Protection*, **14**, 165-169. *****
3. Aslam TJ., Johnson SN. & Karley AJ. (2013). Plant-mediated effects of drought on aphid population structure and parasitoid attack. *Journal of Applied Entomology,* **137**, 136-145. *****
4. Banfield-Zanin JA. & Leather SR. (2015A). Drought intensity and frequency have contrasting effects on development time and survival of the green spruce aphid. *Agricultural and Forest Entomology,* **17**, 309-316. **ǂ**
5. Banfield-Zanin JA. & Leather SR. (2015B). Reproduction of an arboreal aphid pest, *Elatobium abietinum,* is altered under drought stress. *Journal of Applied Entomology,* **139**, 302-313.
6. Banfield-Zanin JA & Leather SR. (2015C). Season and drought stress mediate growth and weight of the green spruce aphid on Sitka spruce. *Agricultural and Forest Entomology,* **17**, 48-56. **ǂ**
7. Barton BT. & Ives AR. (2014). Species interactions and a chain of indirect effects driven by reduced precipitation. *Ecology*, **95**, 486-494. **ǂ**
8. Beetge L. & Kruger K. (2019). Drought and heat waves associated with climate change affect performance of the potato aphid *Macrosiphum euphorbiae.* *Scientific Reports,* **9.** **ǂ***
9. Bultman TL. & Bell GD. (2003). Interaction between fungal endophytes and environmental stressors influences plant resistance to insects. *Oikos*, **103**, 182-190. *****
10. Björkman C .(2000). Interactive effects of host resistance and drought stress on the performance of a gall-making aphid living on Norway spruce. *Oecologia*, **123,** 223-231 *****
11. Björkman C. (1998). Opposite, linear and non-linear effects of plant stress on a galling aphid. Scandinavian *Journal of Forest Research,* ***13*,** 177-183 *****
12. Cabrera HM., Argandona VH., Zuniga GE. & Corcuera LJ. (1995). Effect of infestation by aphids on the water status of barley and insect development. *Phytochemistry*, **40**, 1083-1088. *****
13. Dai P., Liu D. & Shi X. (2015). Impacts of Water Deficiency on Life History of *Sitobion avenae* Clones from Semi-arid and Moist Areas. *Journal of Economic Entomology,* **108**, 2250-2258. **ǂ**
14. Dardeau F., Berthier A., Feinard-Duranceau M., Brignolas F., Laurans F., Lieutier F. & Salle A. (2015). Tree genotype modulates the effects of water deficit on a plant-manipulating aphid. *Forest Ecology and Management*, **353**, 118-125. *****
15. Davis TS., Bosque-Pérez NA., Foote NE., Magney T. & Eigenbrode SD. (2015). Environmentally dependent host–pathogen and vector–pathogen interactions in the *Barley yellow dwarf virus* pathosystem. *Journal of Applied Ecology,* **52**, 1392-1401. *****
16. De Farias AMI., Hopper KR. & Leclant F. (1995). Damage symptoms and abundance of *Diuraphis noxia* (homoptera, aphididae) for 4 wheat cultivars at 3 irrigation levels. *Journal of Economic Entomology*, **88**, 169-174.
17. Fereres A., Gutierrez C., Del Estal P. & Castañera P. (1988). Impact of the English Grain Aphid, *Sitobion avenae* (F.) (Homoptera: Aphididae), on the Yield of Wheat Plants Subjected to Water Deficits. *Environmental Entomology*, **17**, 596-602. *****
18. Foote NE., Davis TS., Crowder DW., Bosque-Perez NA. & Eigenbrode SD. (2017). Plant Water Stress Affects Interactions Between an Invasive and a Naturalized Aphid Species on Cereal Crops. *Environmental Entomology*, **46**, 609-616. **ǂ***
19. Grettenberger IM. & Tooker JF. (2016). Inter-varietal interactions among plants in genotypically diverse mixtures tend to decrease herbivore performance. *Oecologia*, **182**, 189-202. *****
20. Guo H., Sun Y., Peng X., Wang Q., Harris M. & Ge F. (2016). Up-regulation of abscisic acid signaling pathway facilitates aphid xylem absorption and osmoregulation under drought stress. *Journal of experimental botany*, **67**, 681-693. *****
21. Hale BK., Bale JS., Pritchard J., Masters GJ. & Brown VK. (2003). Effects of host plant drought stress on the performance of the bird cherry‐oat aphid, *Rhopalosiphum padi* (L.): a mechanistic analysis. *Ecological Entomology,* **28**, 666-677. **ǂ***
22. Johnson SN., Staley JT., McLeod FAL. & Hartley SE. (2011). Plant-mediated effects of soil invertebrates and summer drought on above-ground multitrophic interactions. *Journal of Ecology*, **99**, 57-65. *****
23. King C., Jacob HS. & Berlandier F. (2006). The influence of water deficiency on the relationship between canola (*Brassica napus* L.), and two aphid species (Hemiptera : Aphididae), *Lipaphis erysimi* (Kaltenbach) and *Brevicoryne brassicae* (L.). *Australian Journal of Agricultural Research*, **57**, 439-445. **ǂ***
24. Larsson S. & Bjorkman C. (1993). Performance of chewing and phloem-feeding insects on stressed trees. *Scandinavian Journal of Forest Research,* **8**, 550-559.
25. Liu D., Dai P., Li S., Ahmed SS., Shang Z. & Shi X. (2018). Life-history responses of insects to water-deficit stress: a case study with the aphid *Sitobion avenae*. BMC Ecology **18**. **ǂ**
26. McVean RIK. & Dixon AFG. (2001). The effect of plant drought-stress on populations of the pea aphid *Acyrthosiphon pisum.* *Ecological Entomology,* **26**, 440-443. *****
27. Mewis I., Khan MAM., Glawischnig E., Schreiner M. & Ulrichs C. (2012). Water Stress and Aphid Feeding Differentially Influence Metabolite Composition in *Arabidopsis thaliana* (L.). *PLoS ONE*, **7**, e48661. **ǂ***
28. Miranda IM., Omacini M. & Chaneton EJ. (2011). Environmental context of endophyte symbioses: Interacting effects of water stress and insect herbivory. *International Journal of Plant Sciences*, **172¸** 499-508. **ǂ***
29. Mody K,. Eichenberger D. & Dorn S. (2009). Stress magnitude matters: different intensities of pulsed water stress produce non-monotonic resistance responses of host plants to insect herbivores. *Ecological Entomology*, **34**, 133-143. *****
30. Moran NA. & Whitham TG. (1988). Population Fluctuations in Complex Life Cycles: An Example from Pemphigus Aphids. *Ecology*, **69**, 1214-1218. **ǂ**
31. Nachappa P., Culkin CT., Saya PM., Han J. & Nalam VJ. (2016). Water Stress Modulates Soybean Aphid Performance, Feeding Behavior, and Virus Transmission in Soybean. *Frontiers in Plant Science,* **7**, 552. *****
32. Neuvonen S., Routio I. & Haukioja E. (1992). Combined effects of simulated acid rain and aphid infestation on the growth of Scots pine (*Pinus sylvestris*) seedlings. *Annales Botanici Fennici,* **29,** 101-106
33. Nguyen TTA., Michaud D. & Cloutier C. (2007). Proteomic profiling of aphid *Macrosiphum euphorbiae* responses to host-plant-mediated stress induced by defoliation and water deficit. *Journal of Insect Physiology*, **53**, 601-611. **ǂ**
34. Oswald CJ. & Brewer MJ. (1997). Aphid-barley interactions mediated by water stress and barley resistance to Russian wheat aphid (Homoptera: Aphididae). *Environmental Entomology,* **26**, 591-602. **ǂ***
35. Pineda A., Pangesti N., Soler R., van Dam NM., van Loon JJA. & Dicke M. (2016). Negative impact of drought stress on a generalist leaf chewer and a phloem feeder is associated with, but not explained by an increase in herbivore-induced indole glucosinolates. *Environmental and Experimental Botany,* **123**, 88-97. *****
36. Pons C., Voß A-C., Schweiger R. & Müller C. (2020). Effects of drought and mycorrhiza on wheat and aphid infestation. *Ecology and Evolution*, **10,** 10481-10491 *****
37. Prill N., Bullock JM., van Dam NM, & Leimu R. 2014. Loss of heterosis and family-dependent inbreeding depression in plant performance and resistance against multiple herbivores under drought stress. *Journal of Ecology,* **102,** 1497-1505. *****
38. Quandahor P., Lin C., Gou Y., Coulter J. & Liu C. (2019). Leaf morphological and biochemical responses of three potato (*Solanum tuberosum* L.) cultivars to drought stress and aphid (*Myzus persicae* Sulzer) infestation. *Insects*, **10**, 435 **ǂ***
39. Ramirez CC. & Verdugo JA. (2009). Water availability affects tolerance and resistance to aphids but not the trade-off between the two. *Ecological Research,* **24**, 881-888.
40. Rivelli A., Trotta V., Toma I., Fanti P. & Battaglia D. (2013). Relation between plant water status and *Macrosiphum euphorbiae* (Hemiptera: Aphididae) population dynamics on three cultivars of tomato. *European Journal of Entomology,* **110**, 617-625. *****
41. Rousselin A., Bevacqua D., Vercambre G., Sauge MH., Lescourret F. & Jordan MO. (2018). Rosy apple aphid abundance on apple is shaped by vegetative growth and water status. *Crop Protection,* **105**, 1-9. *****
42. Rudgers JA. & Swafford AL. (2009). Benefits of a fungal endophyte in *Elymus virginicus* decline under drought stress. *Basic and Applied Ecology,* **10**, 43-51. *****
43. Ryalls JMW., Moore BD., Riegler M. & Johnson SN. (2016). Above-Below ground Herbivore Interactions in Mixed Plant Communities Are Influenced by Altered Precipitation Patterns. *Frontiers in Plant Science*, **7**. *****
44. Simpson KLS., Jackson GE. & Grace J. 2012. The response of aphids to plant water stress – the case of *Myzus persicae* and *Brassica oleracea* var. capitata. *Entomologia Experimentalis et Applicata,* **142**, 191-202. **ǂ***
45. Smyrnioudis IN., Harrington R., Karis N. & Clark SJ. (2000). The effect of drought stress and temperature on spread of barley yellow dwarf virus (BYDV). *Agricultural and Forest Entomology,* **2,** 161-166
46. Tamburini G., van Gils S., Kos M., van der Putten W. & Marini L. (2018). Drought and soil fertility modify fertilization effects on aphid performance in wheat. *Basic and Applied Ecology*, **30**, 23-31. **ǂ***
47. Tariq M., Wright DJ., Bruce TJA & Staley JT. (2013A). Drought and Root Herbivory Interact to Alter the Response of Above-Ground Parasitoids to Aphid Infested Plants and Associated Plant Volatile Signals. *PLoS ONE*, **8**. **ǂ***
48. Tariq M., Rossiter JT., Wright DJ. & Staley JT. (2013B). Drought alters interactions between root and foliar herbivores. *Oecologia*, **172,** 1095-1104. **ǂ***
49. Tariq M., Wright DJ., Rossiter JT. & Staley JT. (2012). Aphids in a changing world: testing the plant stress, plant vigour and pulsed stress hypotheses. *Agricultural and Forest Entomology,* **14**, 177-185. **ǂ***
50. Verdugo JA., Sauge MH., Lacroze JP., Francis F. & Ramirez CC. (2015). Drought-stress and plant resistance affect herbivore performance and proteome: the case of the green peach aphid *Myzus persicae* (Hemiptera: Aphididae). *Physiological Entomology*, **40**, 265-276.
51. Wade RN., Karley AJ., Johnson SN. & Hartley SE. (2017). Impact of predicted precipitation scenarios on multitrophic interactions. *Functional Ecology,* **31**, 1647-1658. **ǂ***
52. Warrington S. & Whittaker JB. 1990. Interactions between sitka spruce, the green spruce aphid, sulfur-dioxide pollution and drought. *Environmental Pollution,* **65**, 363-370. *****
53. Xing GM., Zhang J., Liu J., Zhang XY., Wang GX. & Wang YF. (2003). Impacts of atmospheric CO2 concentrations and soil water on the population dynamics, fecundity and development of the bird cherry-oat aphid *Rhopalosiphum padi.* *Phytoparasitica*, **31**, 499-514. **ǂ***
54. Xie H., Shi J., Shi F., Wang X., Xu H., He K. & Wang Z. (2020). Aphid fecundity and defences in wheat exposed to a combination of heat and drought stress. *Journal of Experimental Botany,* **71**, 2713-2722 *****
55. Yule KM., Woolley JB. & Rudgers JA. (2011). Water availability alters the tri-trophic consequences of a plant-fungal symbiosis. *Arthropod-Plant Interactions,* **5,** 19-27. *****

**Appendix S3:** The number of studies across six geographical regions (A) and the number of studies using controlled environment, field/polytunnel, or glasshouse experimental conditions (B).

**
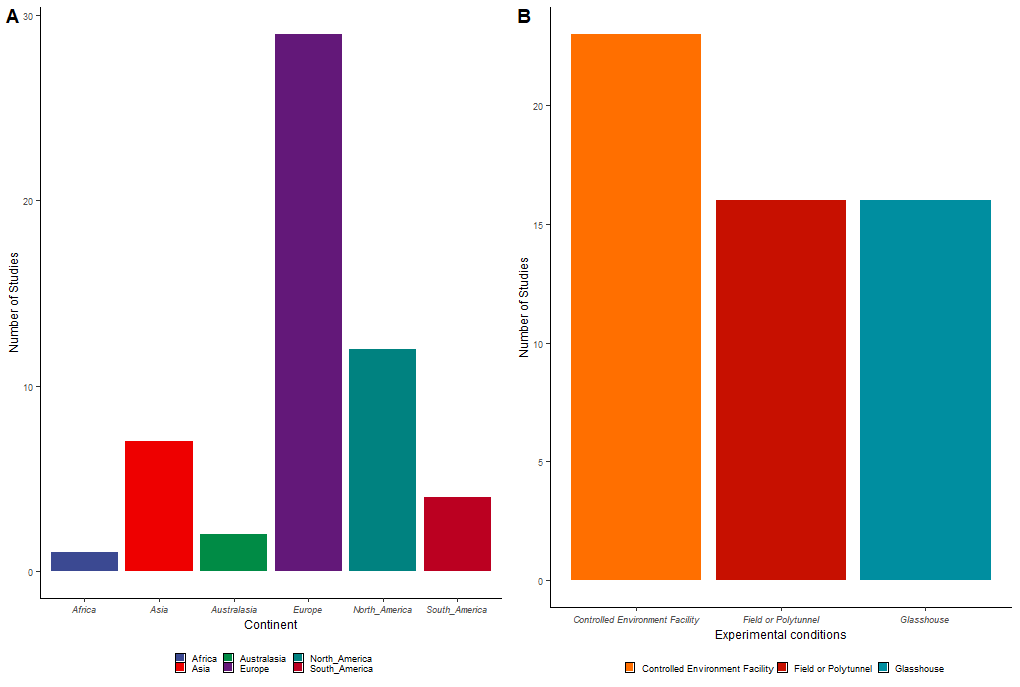
**

**Appendix S4:** The observed effect size for each individual aphid fitness parameter in the “Expanded” dataset coded by the aphid fitness parameter it is categorised into. *N* represents the number of replicates (pooled across treatments to the drought treatment and aphid level) per observed point. Datapoint legend: filled circle – aphid biomass; filled triangle – development; filled square – fecundity; cross – lifespan; crossed open square – population size.

**
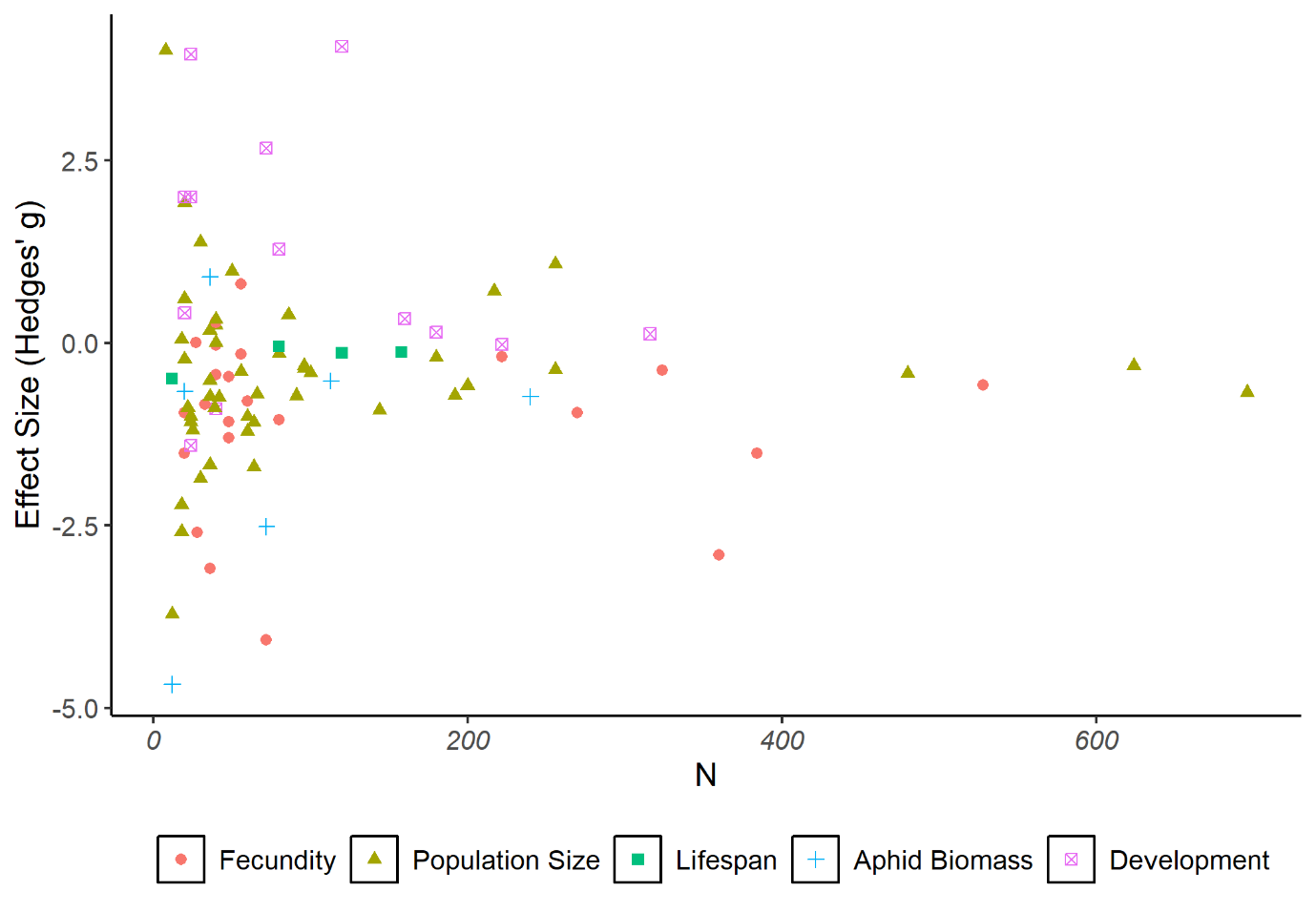
**

**Appendix S5:** The effect of the methodology used to impose drought stress on pooled aphid fitness using the “global” dataset. Drought methodology coding: FC (studies where % reduction in field capacity was used); DI (studies where decreased volume of irrigation was used); GM (studies which used a gravimetric method to adjust irrigation); CC (studies which used a calibration curve to help advise water irrigation regimes); and RW (studies where irrigation was simply restricted or withheld from the drought treated plants). Graph displays the mean effect size (Hedges’ g) and the 95% confidence intervals for the different plant-aphid systems identified from the extracted data. Red dashed line represents zero effect size.

**
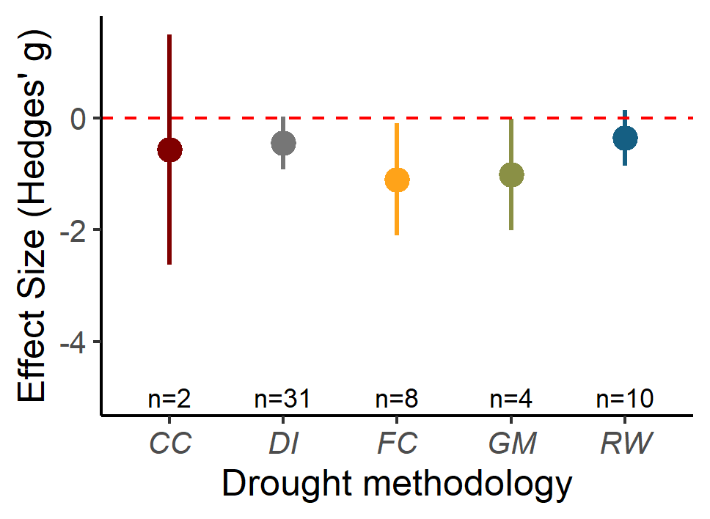
**

**Appendix S6:** Funnel plots for the extracted studies. A) the “global” dataset, B) the “expanded” dataset, C) the dataset used in plant vigour analysis, D) the dataset used in plant nutrition analysis, E) the dataset used in plant defence analysis


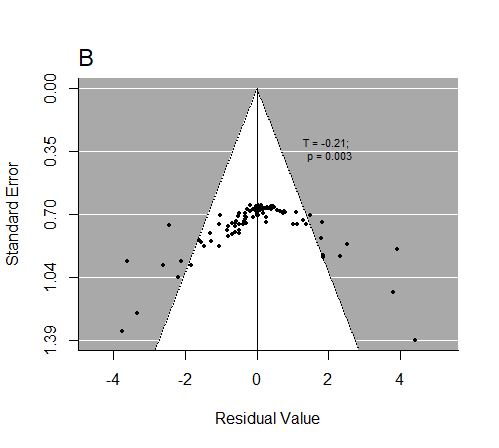

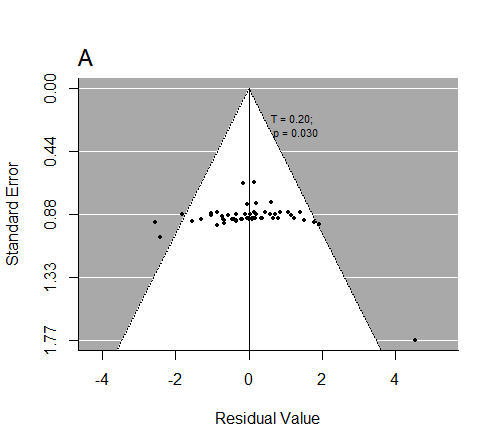

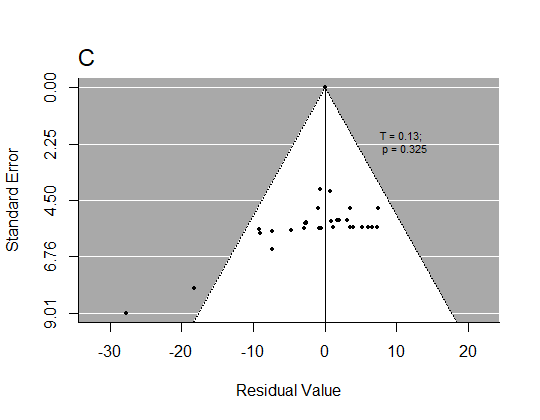

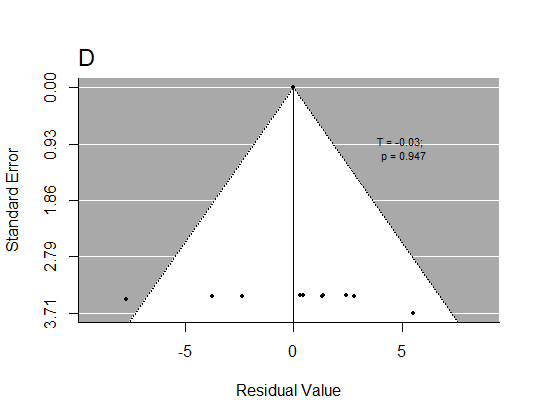

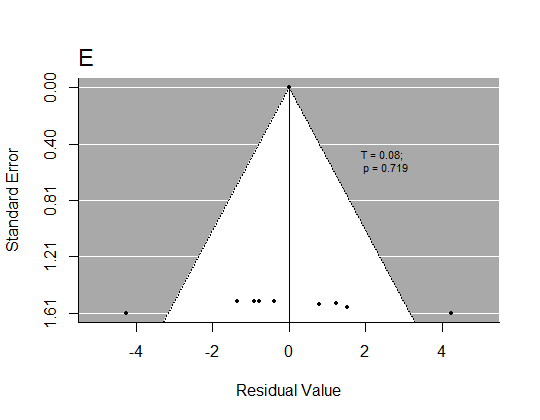


**Appendix S7:** Forest plots for the main meta-analysis dataset.

**A7-1:** Forest plot of the 55 studies included in the meta-analysis of aphid fitness responses to plant drought stress. Plot displays the mean effect size and 95% confidence intervals of pooled aphid responses to drought stress. Blue diamond represents the relative effect size of the model.

**
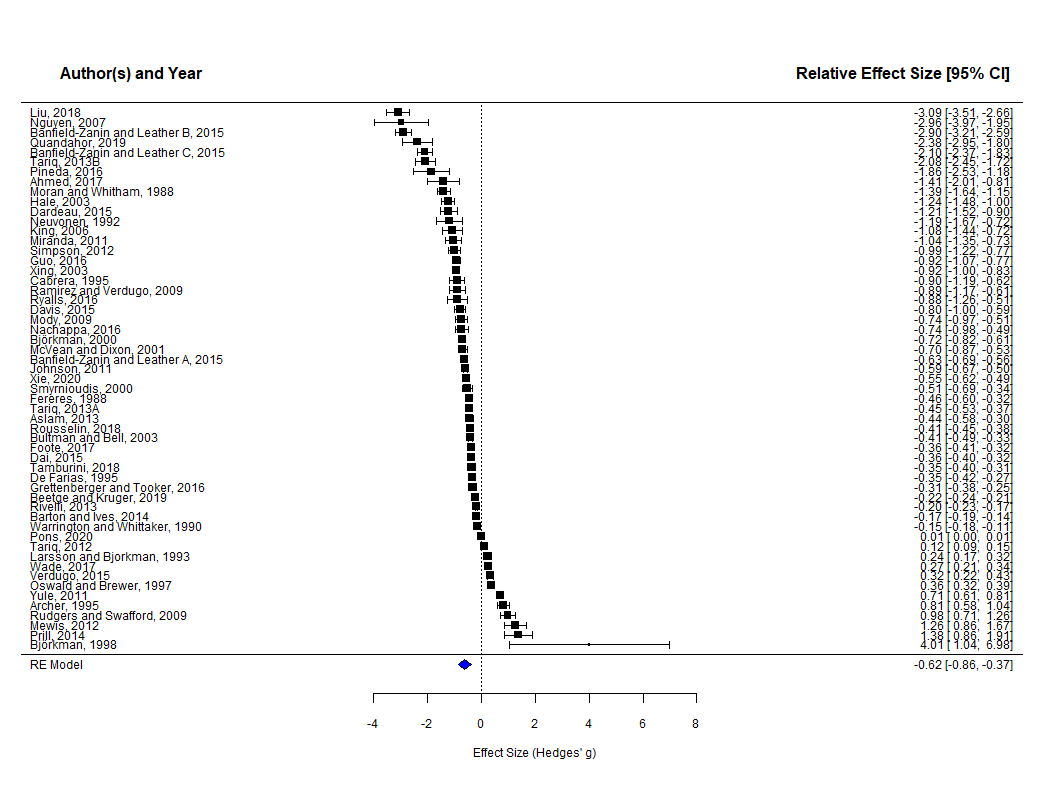
**

**A7-2:** Forest plot of the 32 studies included in the meta-analysis of plant physiological responses to drought stress. Plot displays the mean effect size and 95% confidence intervals. Blue diamond represents the relative effect size of the model.

**
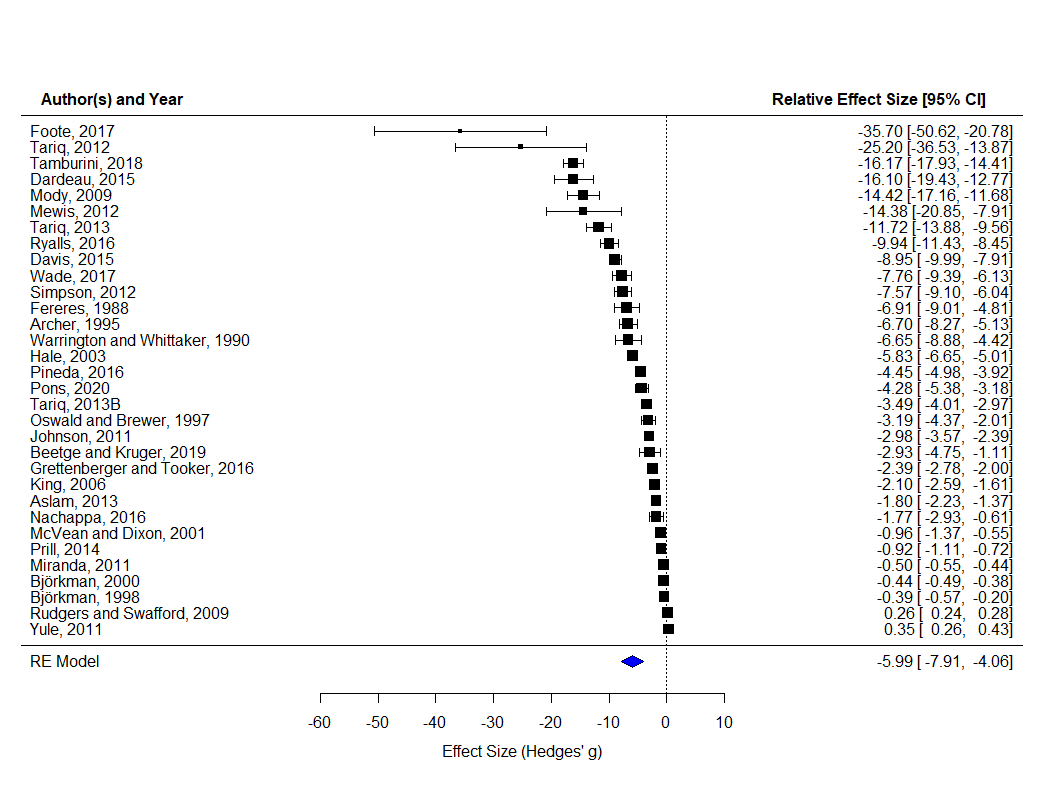
**

**A7-3:** Forest plot of the 12 studies included in the meta-analysis of plant nutritional responses to drought stress. Plot displays the mean effect size and 95% confidence intervals. Blue diamond represents the relative effect size of the model.

**
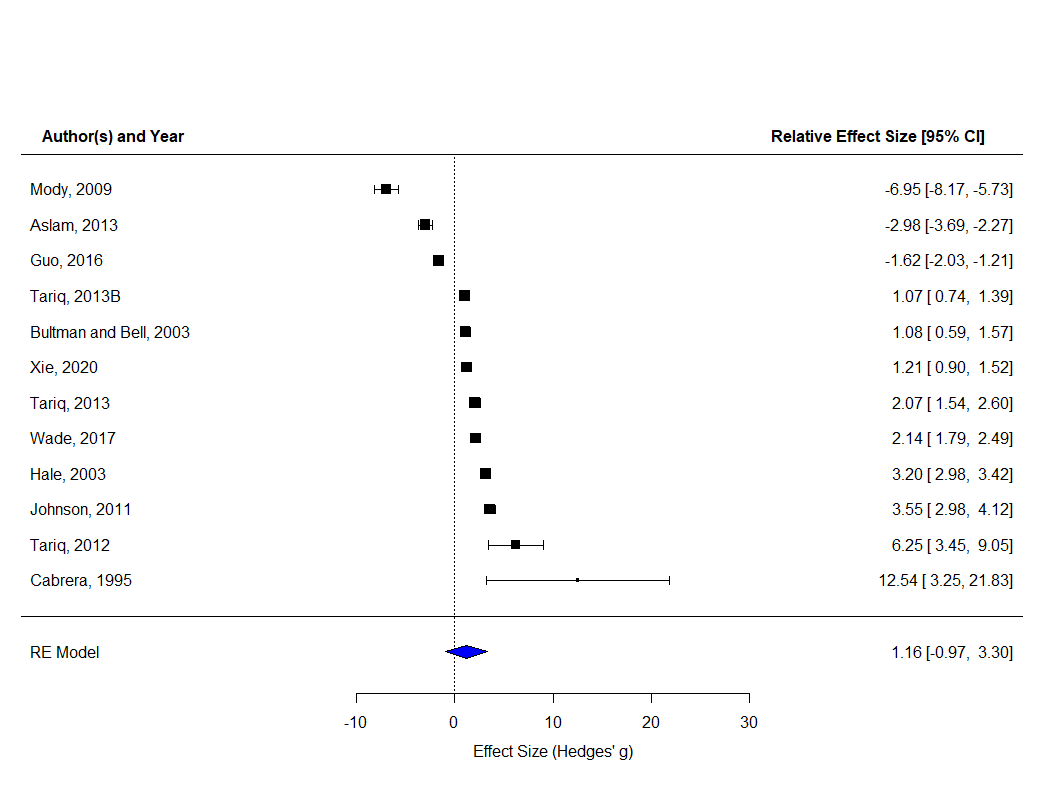
**

**A7-4:** Forest plot of the 7 studies included in the meta-analysis of plant defensive responses to drought stress. Plot displays the mean effect size and 95% confidence intervals. Blue diamond represents the relative effect size of the model.

**
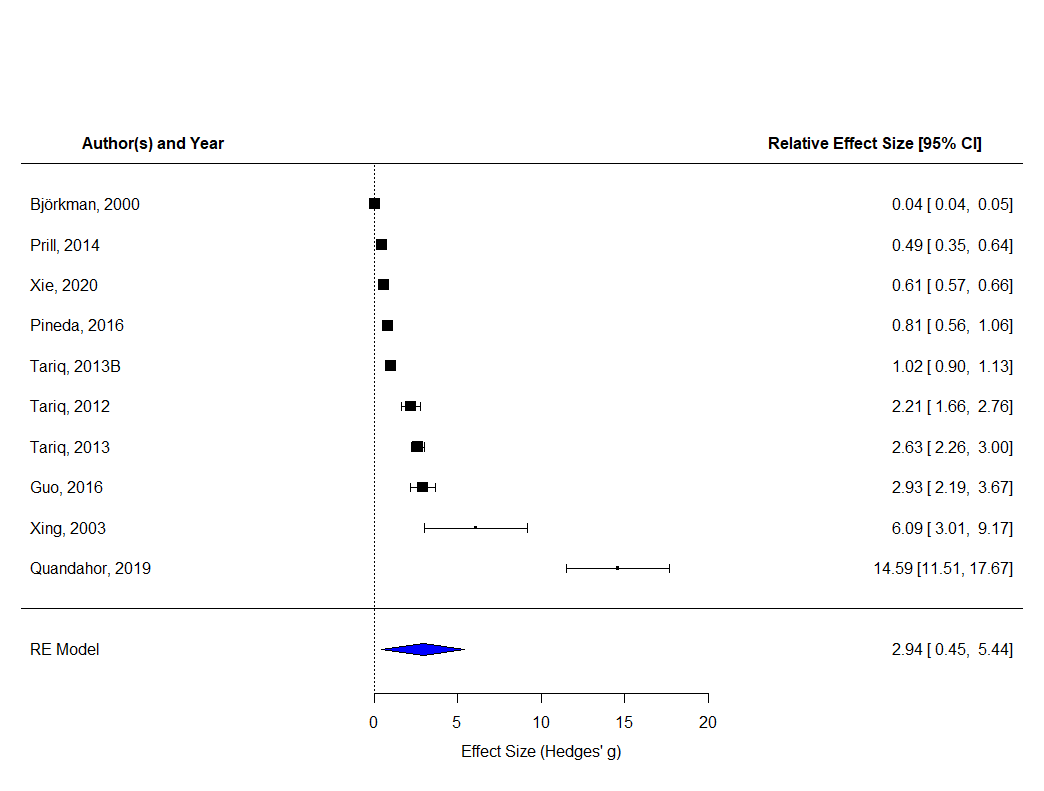
**

**Appendix S8:** Scatter plots showing aphid and plant responses to drought stress over publication time. Red dashed line represents zero effect size.


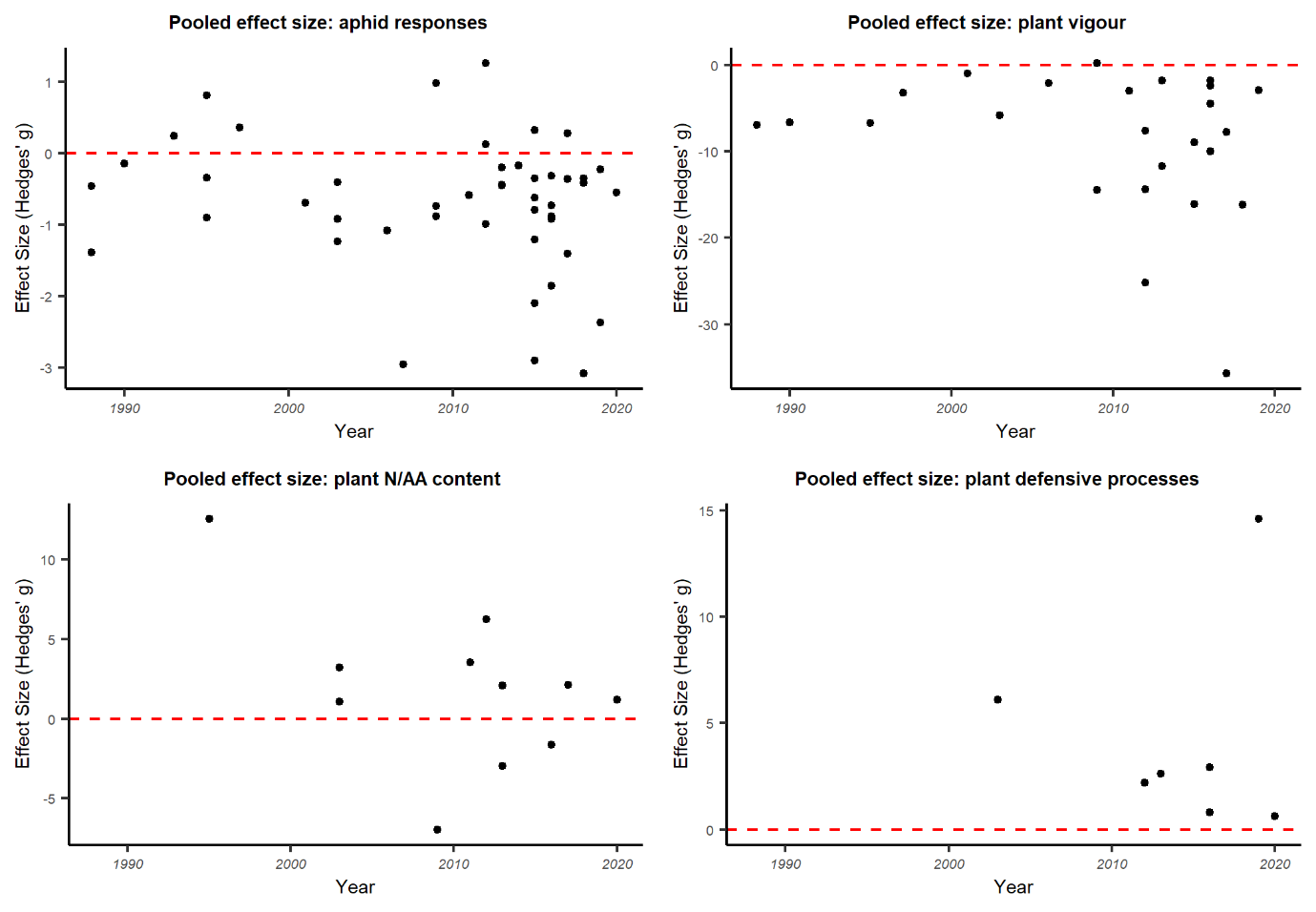


**Appendix S9:** Scatter plots showing the relationship between aphid and plant responses to drought stress. A: The relationship between plant vigour and aphid fitness, B: The relationship between plant N or AA concentration and aphid fitness, C: The relationship between plant defensive compound concentrations and aphid fitness. Red dashed line represents zero effect size for aphid fitness with the dotted red line representing the zero effect size for plant data (vigour, nutritional, defensive).


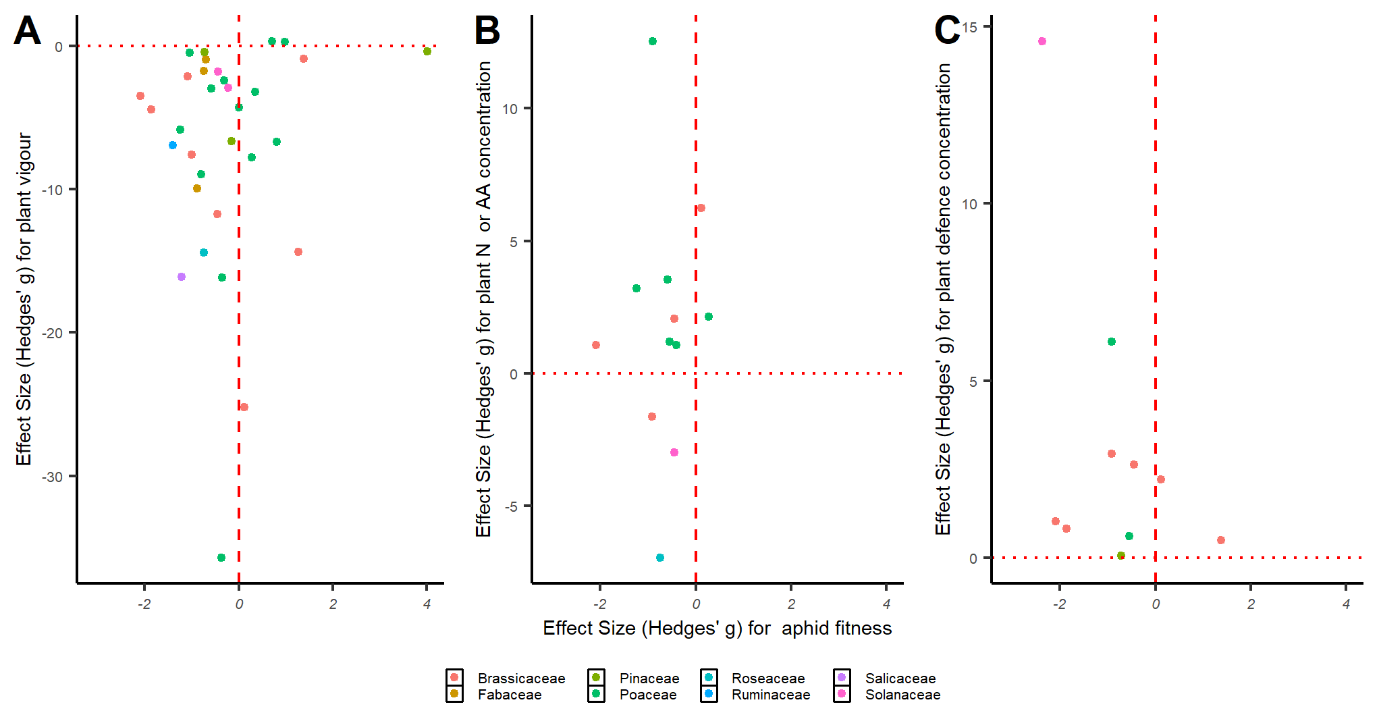

Supplement: Supplementary file 1 — Supplementary Material [file ECE3-11-11915-s001.docx]
